# The neuronal tyrosine kinase receptor ligand ALKAL2 mediates persistent pain

**DOI:** 10.1172/JCI154317

**Published:** 2022-06-15

**Authors:** Manon Defaye, Mircea C. Iftinca, Vinicius M. Gadotti, Lilian Basso, Nasser S. Abdullah, Mélissa Cuménal, Francina Agosti, Ahmed Hassan, Robyn Flynn, Jérémy Martin, Vanessa Soubeyre, Gaetan Poulen, Nicolas Lonjon, Florence Vachiery-Lahaye, Luc Bauchet, Pierre Francois Mery, Emmanuel Bourinet, Gerald W. Zamponi, Christophe Altier

**Affiliations:** 1 Department of Physiology and Pharmacology, Cumming School of Medicine,; 2 Inflammation Research Network–Snyder Institute for Chronic Diseases, Cumming School of Medicine,; 3 Alberta Children’s Hospital Research Institute, and; 4 Hotchkiss Brain Institute, Cumming School of Medicine, University of Calgary, Calgary, Alberta, Canada.; 5 Toulouse Institute for Infectious and Inflammatory Diseases (INFINITy), INSERM UMR1291, CNRS UMR5051, University of Toulouse III, Toulouse, France.; 6 Institute of Functional Genomics, Montpellier University, CNRS, INSERM, Montpellier, France.; 7 Department of Neurosurgery, Gui de Chauliac Hospital, and; 8 Donation and Transplantation Coordination Unit, CHU Montpellier, Montpellier University Medical Center, Montpellier, France.

**Keywords:** Neuroscience, Cancer, Pain, Signal transduction

## Abstract

The anaplastic lymphoma kinase (ALK) is a receptor tyrosine kinase known for its oncogenic potential that is involved in the development of the peripheral and central nervous system. ALK receptor ligands ALKAL1 and ALKAL2 were recently found to promote neuronal differentiation and survival. Here, we show that inflammation or injury enhanced ALKAL2 expression in a subset of TRPV1^+^ sensory neurons. Notably, ALKAL2 was particularly enriched in both mouse and human peptidergic nociceptors, yet weakly expressed in nonpeptidergic, large-diameter myelinated neurons or in the brain. Using a coculture expression system, we found that nociceptors exposed to ALKAL2 exhibited heightened excitability and neurite outgrowth. Intraplantar CFA or intrathecal infusion of recombinant ALKAL2 led to ALK phosphorylation in the lumbar dorsal horn of the spinal cord. Finally, depletion of ALKAL2 in dorsal root ganglia or blocking ALK with clinically available compounds crizotinib or lorlatinib reversed thermal hyperalgesia and mechanical allodynia induced by inflammation or nerve injury, respectively. Overall, our work uncovers the ALKAL2/ALK signaling axis as a central regulator of nociceptor-induced sensitization. We propose that clinically approved ALK inhibitors used for non–small cell lung cancer and neuroblastomas could be repurposed to treat persistent pain conditions.

## Graphical abstract

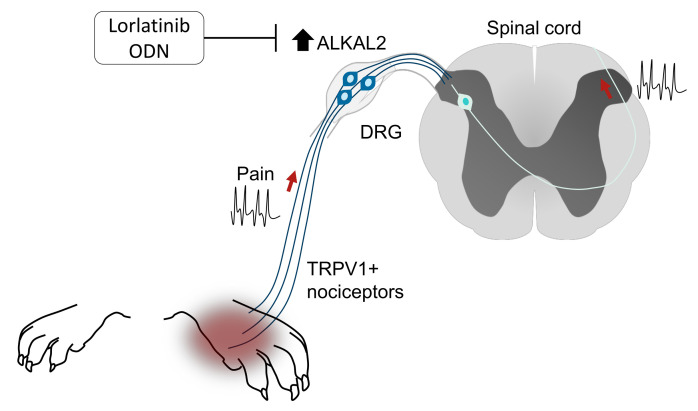

## Introduction

Sensitization of nociceptive neurons can lead to persistent pain in response to inflammation or injury. Identifying the mechanisms of peripheral sensitization has been key to defining the maladaptive long-lasting changes in nociceptive circuits that ultimately precipitate the transition to chronic pain (1–3). During inflammation, this sensitization process occurs particularly in thinly myelinated Aδ and unmyelinated C-fibers that express the transient receptor potential vanilloid 1 (TRPV1) channel (2–5) and transduce inflammatory stimuli. Despite characterization of a large number of inflammatory mediators, their receptors, and downstream signaling pathways, very few of these targets have led to efficacious treatments for pain relief (6, 7).

In the search for new therapeutics, targeting the receptor tyrosine kinase (RTK) family has shown promise for persistent pain conditions (6–11). Two members of the RTK family are the leukocyte tyrosine kinase (LTK) and the anaplastic lymphoma kinase (ALK), which belong to the insulin receptor family (12–15). Both members of this subfamily have been reported as promoting cellular differentiation and growth, yet the physiological role of ALK has remained unclear. Initially described for its oncogenic properties (such as its connection with large B cell lymphoma, non–small cell lung carcinoma [NSCLC], or neuroblastoma; refs. 16, 17), ALK was recently found to play an important role in the developing nervous system (18–20).

As aberrant activation of ALK is involved in malignancy, research has focused on developing specific inhibitors for cancer therapy, and many of them are now used in clinical practice (21–23). Crizotinib (Xalkori, Pfizer) represents the first generation of FDA-approved ALK inhibitor. However, crizotinib had poor specificity, acting on ROS proto-oncogene 1–encoded kinase (ROS1), and was unable to penetrate the blood-brain barrier. More recently, the third-generation molecule lorlatinib (PF-06463922) was introduced to overcome crizotinib resistance. Lorlatinib has higher potency than previous generations of ALK inhibitors and shows good penetration of the blood-brain barrier (24).

As a classical RTK, ALK is composed of an extracellular domain, a single transmembrane domain, and a cytoplasmic domain that phosphorylates and activates multiple intracellular signaling pathways, driving neuronal differentiation (25, 26). Recently, 2 novel proteins, family with sequence similarity 150A (FAM150A or ALKAL1) and family with sequence similarity 150B (FAM150B, ALKAL2), were identified as high-affinity agonists of ALK/LTK receptors (26–28). Interestingly, ALKAL1 and ALKAL2 display homology only with one another and appear in several mammalian tissues, including rat nerves, where they induce the rapid formation of processes (14, 26, 27). Although both proteins physically interact with ALK, ALKAL1 shows greater affinity for LTK and ALKAL2 toward ALK (27, 28).

Here, we identified ALKAL2, the ligand for the RTK ALK, as a biomarker of inflammation-induced nociceptor sensitization. We examined its pronociceptive properties and tested the analgesic efficacy of the clinically available ALK inhibitors crizotinib and lorlatinib in mouse models of inflammatory and neuropathic pain.

## Results

### ALKAL2 is upregulated in TRPV1 nociceptors during inflammation.

**Figure 1:**
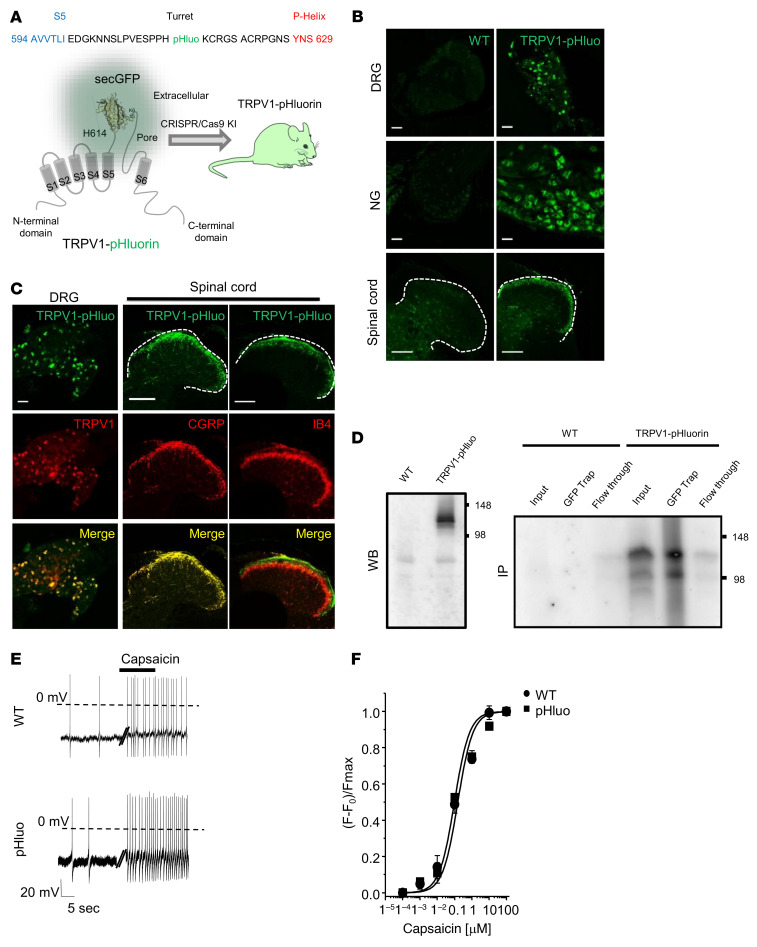
Characterization of the TRPV1-pHluorin knockin mouse. (**A**) PHluorin was inserted into the turret region of the TRPV1 channel, between residues H614 and K615 using CRISPR/Cas9 technology (31, 32). (**B**) Representative confocal images showing GFP expression in DRG, nodose ganglion (NG), and spinal cords of WT and TRPV1-pHluorin mouse. Scale bars: 50 μm (DRG and nodose ganglion); 100 μm (spinal cord). (**C**) Coimmunostaining of GFP and TRPV1 in DRG; coimmunostaining of GFP with markers of peptidergic (CGRP) or nonpeptidergic (IB4) neurons in the spinal dorsal horn. Scale bars: 50 μm (DRG); 100 μm (spinal cord). (**D**) Western blot of DRG lysates from WT and TRPV1-pHluorin mice using an anti-GFP antibody; note the specific band at approximately 125 kDa only in the transgenic mice. Immunoprecipitation of TRPV1-pHluorin from DRG lysates using GFP-trap. Membranes were then probed with an anti-TRPV1 antibody. Note the absence of band in WT animals. (**E**) Representative whole-cell current-clamp recordings of the capsaicin-evoked AP discharge in DRG neurons from WT and TRPV1-pHluorin mice. (**F**) Dose-response curve evoked by capsaicin, measured by calcium imaging on DRG neurons from WT (circles) (EC_50_ = 0.91 ± 0.12 μM, *n* = 34) or TRPV1-pHluorin (pHluo) mice (squares) (EC_50_ = 1.06 ± 0.09 μM, *n* = 28) at RT (22°C). Data are represented as mean ± SEM.

TRPV1 expression has been described in nonpeptidergic and myelinated nociceptors earlier during development (29). Therefore, rather than using a lineage-tracing approach with TRPV1-Cre mice, we created a TRPV1-pHluorin knockin mouse to identify the differentially expressed genes in TRPV1 nociceptive neurons. In this mouse, TRPV1 was conjugated to the super-ecliptic pHluorin inserted into the extracellular turret region of the channel (Figure 1A and ref. 30).

Insertion of pHluorin was previously found to not compromise the expression, trafficking, and sensitivity of the channel to capsaicin (31, 32). In addition, heat and pH sensitivity of TRPV1-pHluorin dorsal root ganglia (DRG) neurons were identical to those in the WT channel (Supplemental Figure 1; supplemental material available online with this article; https://doi.org/10.1172/JCI154317DS1). We next examined the expression of TRPV1-pHluorin in the DRG and the nodose/jugular ganglia (Figure 1B) and in central terminals of nociceptors located in the superficial dorsal horn of the spinal cord (Figure 1, B and C). The molecular identity of TRPV1^+^ neurons was verified by colabeling analysis with calcitonin gene-related peptide (CGRP) (peptidergic nociceptors), IB4 (nonpeptidergic), and TH (C-LTMR) (Supplemental Figure 2). The number and size of TRPV1^+^ neurons in DRGs and the innervation pattern in the spinal dorsal horn (Figure 1, B and C, and Supplemental Figure 2) were not altered in the TRPV1-pHluorin mouse. Moreover, TRPV1-pHluorin protein could be immunoprecipitated from DRG tissue extracts, using GFP beads that recognize the pHluorin variant of GFP (Figure 1D). We then validated the functional expression of TRPV1-pHluorin, previously reported in heterologous expression systems (33), in mouse DRGs. Capsaicin evoked action potential (AP) firing in pHluorin-labeled neurons (Figure 1E), with an EC_50_ similar to that for the WT channel (Figure 1F). Next, we performed FACS purification of TRPV1-pHluorin neurons isolated from adult mouse DRGs pooled from the lumbar region, using a gating of GFP^hi^ cell population. RNA was extracted from GFP^+^ and GFP^–^ neurons, and quantitative PCR (qPCR) was run for specific markers of TRPV1 nociceptors (34), including TRPV1, MOR (mu opioid receptor), or Tac1 (substance P), which were found to be enriched in GFP^+^ TRPV1 neurons relative to GFP^–^ neurons (Supplemental Figure 2E). These results indicate that the molecular signature and neuroanatomical organization of TRPV1 neurons are conserved in the TRPV1-pHluorin knockin mouse.

**Figure 2:**
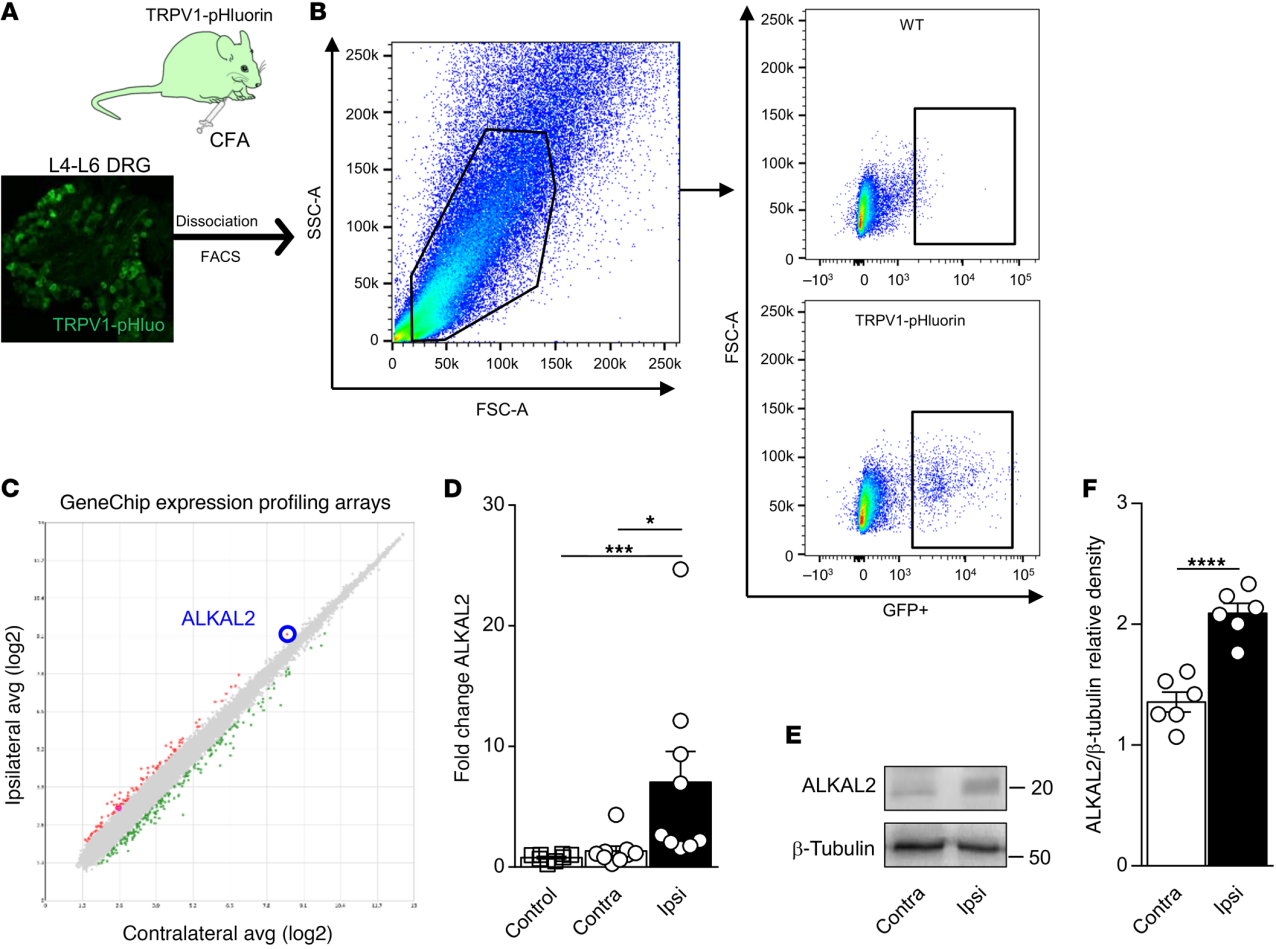
CFA inflammation induces ALKAL2 upregulation in TRPV1 neurons. (**A**) Experimental approach used to conduct the microarray analysis from TRPV1 neurons, 72 hours after i.pl. CFA. (**B**) FACS isolation of TRPV1-pHluorin neurons. Representative FACS plot of GFP^+^ population in WT (top) and TRPV1-pHluorin (bottom) mice. SSC-A side scatter area; FSCA- forward scatter area. (**C**) Scatter plot representation of genes regulated in CFA conditions. Genes that passed a threshold of log_2_ fold change in differential expression analysis are represented as green when downregulated and red when upregulated. All genes are listed in Supplemental Table 1. (**D**) qRT-PCR assessment of ALKAL2 upregulation in the DRG ipsilateral to the CFA injection (Ipsi) (*n* = 9), compared with the contralateral side (Contra) (*n* = 9) and naive control (*n* = 8). Statistical analysis was performed using Kruskal-Wallis followed by Dunn’s post hoc test. *P < 0.05; ***P < 0.001. (**E**) Representative Western blot of ALKAL2 in the DRG ipsilateral to the CFA injection compared with the contralateral side. (**F**) Quantification of ALKAL2 protein level from Western blot experiments. Each dot represents a sample collected from a different animal (*n* = 6 per group). Statistical analysis was performed by unpaired *t* test (**F**). ****P < 0.0001. Data are represented as mean ± SEM.

**Figure 3:**
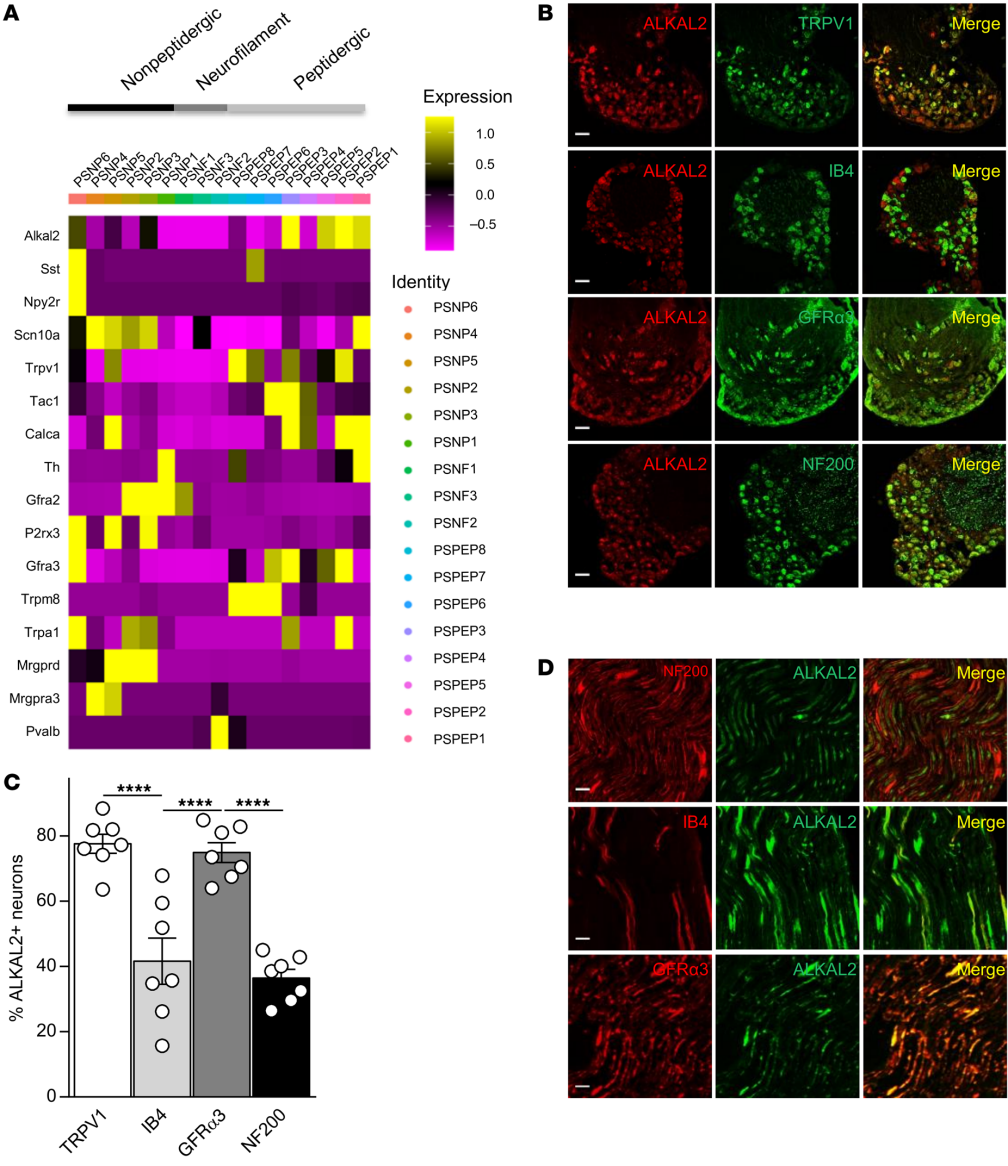
Characterization of mouse ALKAL2 expression. (**A**) Heatmap of the expression of ALKAL2 and selected population markers on the 17 populations of sensory neurons from DRG described in Zeisel et al. (35). (**B**) Representative confocal images of coimmunostaining for ALKAL2 and TRPV1, IB4, GFRα3, and NF200 in DRG neurons. Scale bars: 50 μm. (**C**) Dot plot summarizing the results in **B**: 77.64% ± 2.95% of TRPV1, 77.64% ± 3.06% of GFRα3, 41.65% ± 7.08% of IB4, and 36.46% ± 2.64% of NF200-positive neurons express ALKAL2 (each symbol represents a DRG section from *n* = 4 individual animals). Statistical analysis was performed using 1-way ANOVA followed by Tukey’s post hoc test. ****P < 0.001. (**D**) Representative confocal images of coimmunostaining for ALKAL2 and NF200, IB4, and GFRα3 in the sciatic nerve. Scale bars: 50 μm. Data are represented as mean ± SEM.

**Figure 4:**
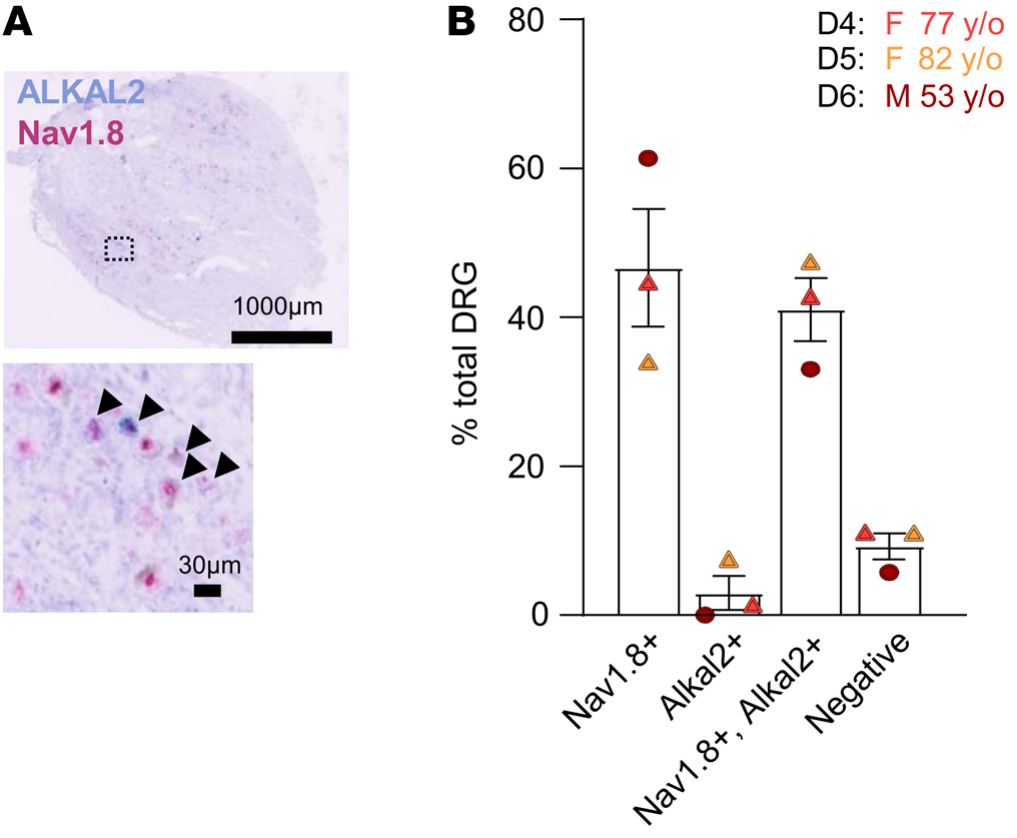
ALKAL2 expression in human DRGs. (**A**) Representative RNAScope image showing expression of ALKAL2 (light blue) in human DRG neurons coexpressing Nav1.8 (pink). (**B**) Bar graph summarizing the results (each symbol represents an individual patient, *n* = 3). Data are represented as mean ± SEM.

To identify putative genes that could contribute to the phenotypic plasticity of TRPV1 nociceptors in inflammation-induced sensitization, we used the CFA model of chronic inflammatory pain (Figure 2A). Three days after intraplantar (i.pl.) injection of CFA, ipsilateral and contralateral lumbar (L4–L6) DRG neurons were separated, and TRPV1-pHluorin neurons were purified by FACS and analyzed through the GeneChip Mouse Gene 2.0 ST Array (Figure 2B). The array analysis provided a complete expression profile of mRNA between contralateral and ipsilateral TRPV1 neurons following inflammatory insult (Figure 2C). Under inflammatory conditions, we found more than 100 genes to be up- or downregulated in TRPV1 neurons (Supplemental Table 1), and among them, we noticed an increase in ALKAL2 (FAM150B) when compared with the contralateral side (Figure 2C). Using qPCR, we measured an approximately 7-fold increase in ALKAL2 mRNA from CFA-injected ipsilateral DRGs relative to either contralateral or control DRGs from PBS-injected mice (Figure 2D). We next assessed ALKAL2 protein levels using an epitope-directed antibody. A band with a MW of approximately 22 kDa (expected size of approximately 15 kDa) was detected by Western blot analysis from DRG lysates (Figure 2E). Importantly, production of ALKAL2 was enhanced in the ipsilateral DRGs of CFA-injected mice (Figure 2F). Evaluation of ALKAL2 expression in a mouse single-cell RNA-Seq data set from the Linnarsson lab (35) identified ALKAL2 in the majority of sensory neurons (Supplemental Figure 3A). To confirm the transcriptomic data, we assessed and compared ALKAL2 protein levels in the cortex, hippocampus, basal ganglia, spinal cord, and DRG (Supplemental Figure 3B). Notably, the intensity of the signal was greater in the DRGs compared with the CNS (Supplemental Figure 3C), confirming the high expression level of ALKAL2 in sensory neurons. Analysis of the scRNA-Seq data set of mouse DRG neurons from Zeisel et al. (35) showed that ALKAL2 was preferentially expressed with nociceptor-specific transcripts, including Trpv1, Tac1 (substance P), Calca (CGRP), GFRα3, and Trpa1 (Figure 3A). We thus performed immunostaining to determine the levels of ALKAL2 protein in the different subpopulations of DRG neurons: TRPV1, IB4 (nonpeptidergic), GFRα3 (peptidergic), and NF200 (large, myelinated neurons). As indicated by the transcriptomic and qPCR data, ALKAL2 was found in peptidergic TRPV1 neurons (~80%), validating the microarray data of Figure 2, and to a lesser extent in nonpeptidergic and NF200^+^ neurons (Figure 3, B and C). Accordingly, in the sciatic nerve, ALKAL2^+^ fibers were clearly identified in GFRα3^+^ peptidergic neurons and did not overlap with myelinated NF200^+^ fibers, again confirming the expression of ALKAL2 in unmyelinated C fibers (Figure 3D). Importantly, we also evaluated the expression of ALKAL2 in human DRG neurons and spinal cord by RNAScope. We used probes for mRNAs encoding Nav1.8 to identify nociceptors (Figure 4A) and found a high degree of colocalization between ALKAL2 and Na_v_1.8^+^ nociceptors (Figure 4B). In contrast, mRNA-encoding ALKAL2 was not found in the human spinal cord (Supplemental Figure 4), indicating that it is selectively expressed and produced in afferent fibers.

### ALKAL2 enhances neurite outgrowth and excitability via ALK.

**Figure 5:**
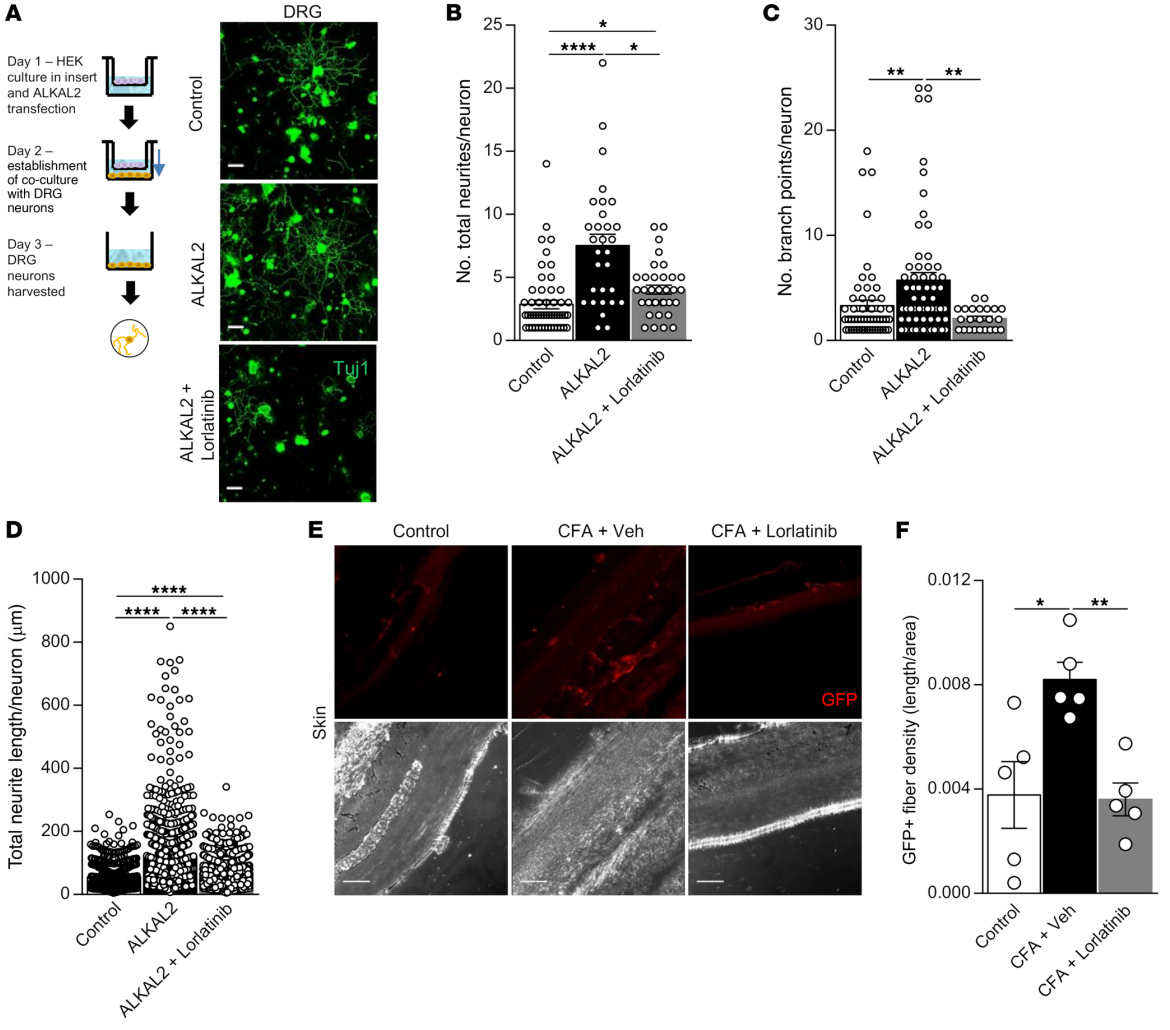
ALKAL2 induces sprouting of DRG neurons. (**A**) Schematic illustrating the coculture system used to chronically expose DRG neurons to ALKAL2. HEK cells were plated into the upper chamber of a Transwell and then transfected with ALKAL2 plasmid for 16 hours. Cells were washed, and DRG neurons were plated in the lower chamber of the Transwell for another 16 hours of coculture and then immunostained for Tuj1. Scale bars: 50 μm. ALKAL2 induces a significant increase of total neurites (**B**), number of branch points per neuron (**C**), and total neurite length per neuron (**D**) (<20 μm). Control, *n* = 19; ALKAL2, *n* = 12; ALKAL2+lorlatinib, *n* = 18. Statistical analysis was performed using Kruskal-Wallis followed by Dunn’s post hoc test. *P < 0.05; **P < 0.01; ****P < 0.001. (**E**) Representative confocal images illustrating the TRPV1-GFP innervation of the skin paw following i.pl. injection of CFA (3 days). Scale bars: 50 μm. (**F**) CFA-induced sprouting is reversed by daily administration of lorlatinib (1 mg/kg). Control, *n* = 5; CFA+vehicle, *n* = 5; CFA+lorlatinib, *n* = 5. Statistical analysis was performed using 1-way ANOVA followed by Tukey’s post hoc test. *P < 0.05; **P < 0.01. Data are represented as mean ± SEM.

Previous work has shown that the ALK receptor plays a central role in the development of neural crest–derived cells and neurite outgrowth (18, 19, 36, 37). Using a coculture system, we tested to determine whether secreted ALKAL2 is able to activate ALK on DRG neurons, thus promoting neurite outgrowth. In this assay, HEK cell monolayers seeded in the upper chamber of a Transwell were transfected with ALKAL2 and then placed above acutely dissociated DRG neurons seeded into the lower chamber (Figure 5A). DRG neurons were exposed to HEK-secreted ALKAL2 for a further 16 hours, after which neurite growth was assessed on small and medium size neurons (Figure 5A). In the ALKAL2-transfected conditions, we measured a 2-fold increase in the total number of neurites (both primary and secondary branches) as well as branching points and a 5-fold increase in the total neurite length per neuron (Figure 5, B–D). Pharmacological inhibition of ALK with lorlatinib was able to reduce ALKAL2-mediated neurite outgrowth, supporting a neurotrophic role of ALKAL2/ALK signaling in sensory neurons. In vivo, we observed an ALK-dependent increase of TRPV1-GFP^+^ fibers in the footpad of CFA-treated mice, which was blocked by lorlatinib (Figure 5, E and F). This indicated that ALK activation contributes to nerve sprouting caused by inflammation.

**Figure 6:**
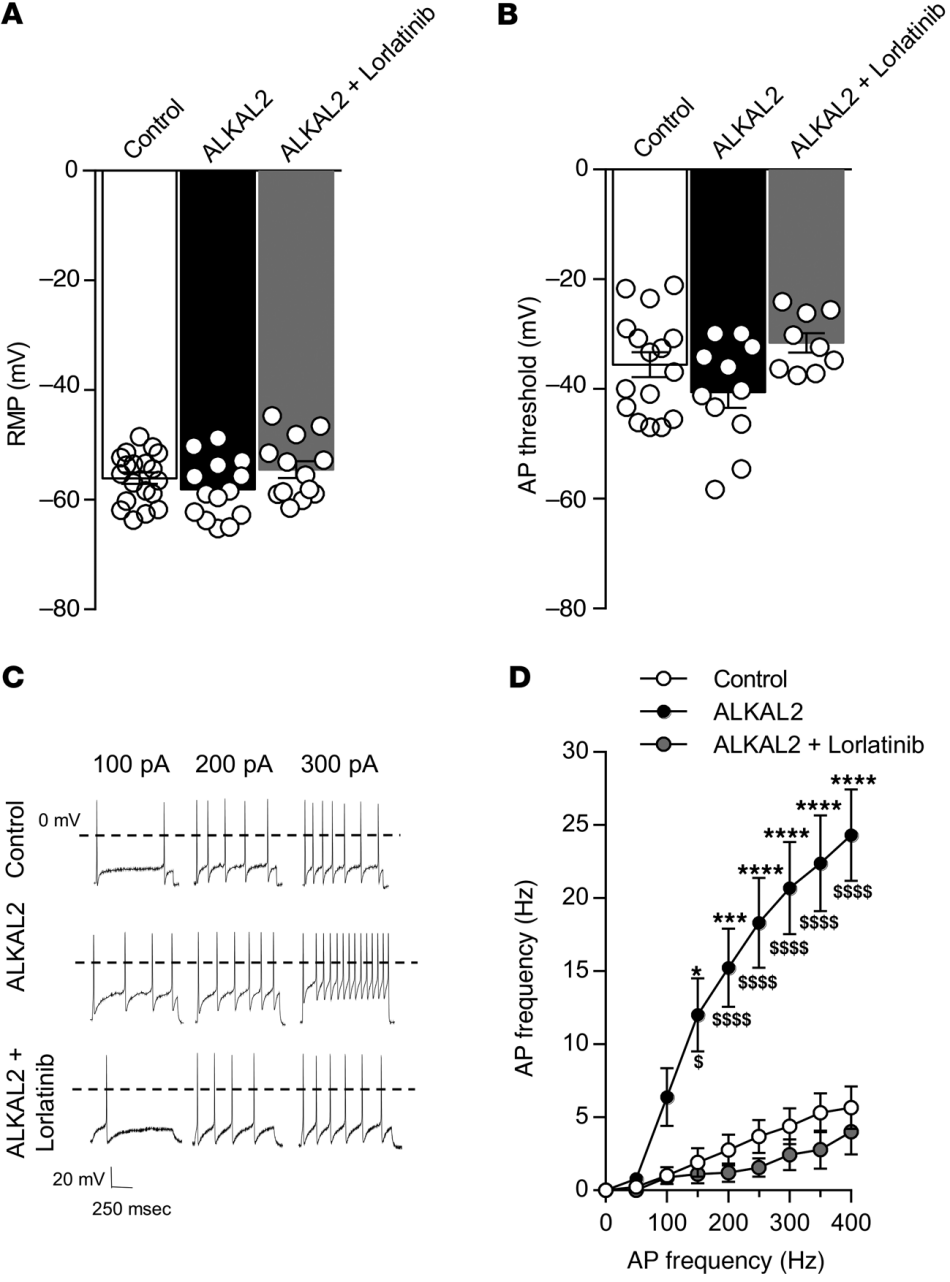
ALKAL2 induces hyperexcitability of small DRG neurons. (**A**) Resting membrane potential (RMP) of small DRG neurons in control (–56.06 ± 1.03 mV, *n* = 19), ALKAL2 (–55.15 ± 2.21 mV, *n* = 13), or ALKAL2+lorlatinib (–54.5 ± 1.53 mV, *n* = 13) groups. Statistical analysis was performed using 1-way ANOVA followed by Tukey’s post hoc test. (**B**) AP threshold in control (–35.59 ± 2.26 mV, *n* = 16), ALKAL2 (–40.57 ± 2.88 mV, *n* = 11), and ALKAL2+lorlatinib (–31.59 ± 1.76 mV, *n* = 9) groups. Statistical analysis was performed using 1-way ANOVA followed by Tukey’s post hoc test. (**C**) Representative AP discharge evoked by 100, 200, 300, and 400 pA current injections (1 s) in control, ALKAL2- (10 nM), and ALKAL2+lorlatinib-treated (10 nM+1 μM) DRG neurons. (**D**) Measure of AP frequency evoked by current injection in the different groups represented in **E** (control, *n* = 13; ALKAL2 [10 nM], *n* = 11; ALKAL2+lorlatinib treated [10 nM+1 μM], *n* = 9). Statistical analysis was performed using 2-way ANOVA followed by Tukey’s post hoc test. *P < 0.05; ***P < 0.001; ****P < 0.0001 versus control. ^$^*P* < 0.05; ^$$$$^*P* < 0.0001 versus ALKAL2+lorlatinib. Data are represented as mean ± SEM.

To determine whether ALKAL2-mediated neurite growth was associated with neuronal hyperexcitability, we assessed the electrophysiological properties of TRPV1 neurons in response to ALKAL2. Neurons exposed to recombinant ALKAL2 for 16 hours did not exhibit a change in their resting membrane potential or their AP threshold (Figure 6, A and B). However, ALKAL2 induced a pronounced increase in AP frequency evoked by ascending steps of injected current (Figure 6, C and D), and this effect was reversed by adding lorlatinib into the DRG cell culture media. This finding suggests that ALKAL2 can promote neurite elongation along with hyperexcitability of TRPV1 neurons through an ALK receptor–mediated mechanism.

### Activation of ALK by ALKAL2 induces hyperalgesia.

**Figure 7:**
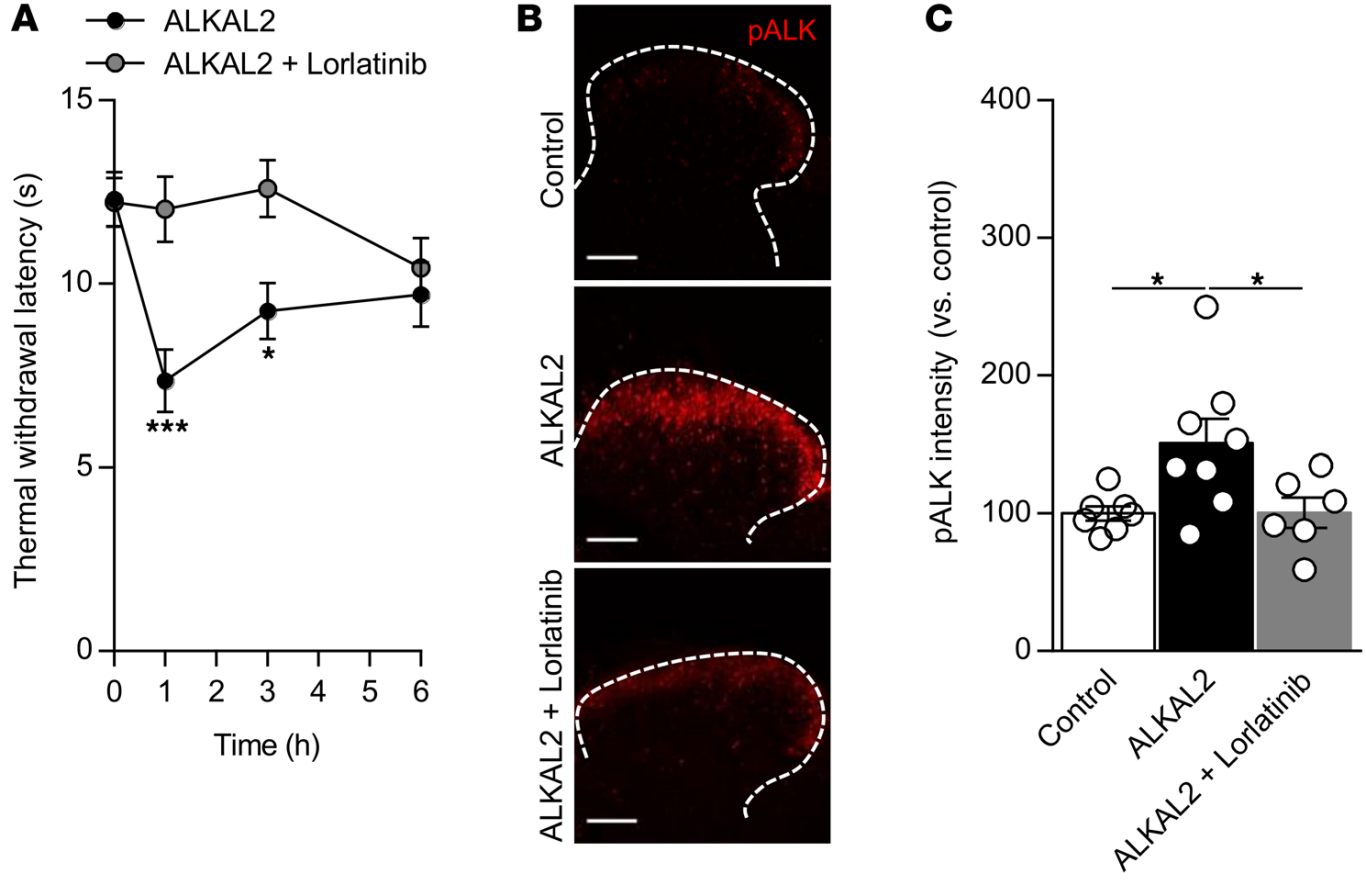
ALKAL2 induces pALK in the dorsal horn of the spinal cord. (**A**) Administration i.t. of ALKAL2 (1 μM) promotes thermal hyperalgesia, which is reversed by the administration of lorlatinib (ALKAL2, *n* = 9; ALKAL2+lorlatinib, *n* = 8). Statistical analysis was performed using 2-way ANOVA followed by Bonferroni’s post hoc test. *P < 0.05; ***P < 0.001. (**B**) Representative confocal images illustrating pALK induction in the lamina I and II of the spinal dorsal horn following i.t. infusion of ALKAL2. Activation of pALK is reversed by administration of lorlatinib prior to ALKAL2 administration. Scale bars: 100 μm. (**C**) Bar graph of the pALK signal intensity represented in **B** in the spinal dorsal horn (control, *n* = 7; ALKAL2, *n* = 8; ALKAL2+lorlatinib, *n* = 6; 1 hour). Statistical analysis was performed using 1-way ANOVA followed by Tukey’s post hoc test. *P < 0.05. Data are represented as mean ± SEM.

To investigate whether ALKAL2 could induce pain in vivo, we administered recombinant ALKAL2 by intrathecal (i.t.) injection, thereby targeting DRG and spinal cord neurons. In naive mice, ALKAL2 induced thermal hyperalgesia lasting several hours in a dose-dependent manner (Supplemental Figure 5A). At a concentration of 1 μM of ALKAL2, thermal hyperalgesia was blocked by a coadministration of lorlatinib (Figure 7A). Immunohistochemical analysis of the activated receptor in the spinal dorsal horn showed an increase in pALK 30 minutes following ALKAL2 injection (Figure 7, B and C). Notably, activation of ALK extends dorsally to the superficial lamina I that responds to noxious stimuli and lamina II projection neurons that contribute to mechanical allodynia (ref. 38 and Figure 7B). Importantly, oral administration of lorlatinib 1 hour before ALKAL2 injection blocked ALK receptor activation (Figure 7, B and C). Thus, ALKAL2 is able to induce ALK activation in the spinal cord, which leads to hyperalgesia.

Next, we assessed the activation of the ALK receptor in the inflammatory pain model (Supplemental Figure 5B). i.pl. CFA enhanced the pALK signal in the ipsilateral (CFA injected) spinal dorsal horn, whereas no changes were observed in saline-injected mice (Supplemental Figure 5C). Importantly, the pattern of ALK activation in the superficial laminae I and II was similar to the one obtained in response to ALKAL2 injection. Thus, ALKAL2-induced hyperalgesia is mediated by the activation of ALK in the spinal dorsal horn.

### ALKAL2 depletion or pharmacological inhibition of ALK prevents persistent inflammatory and neuropathic pain.

**Figure 8:**
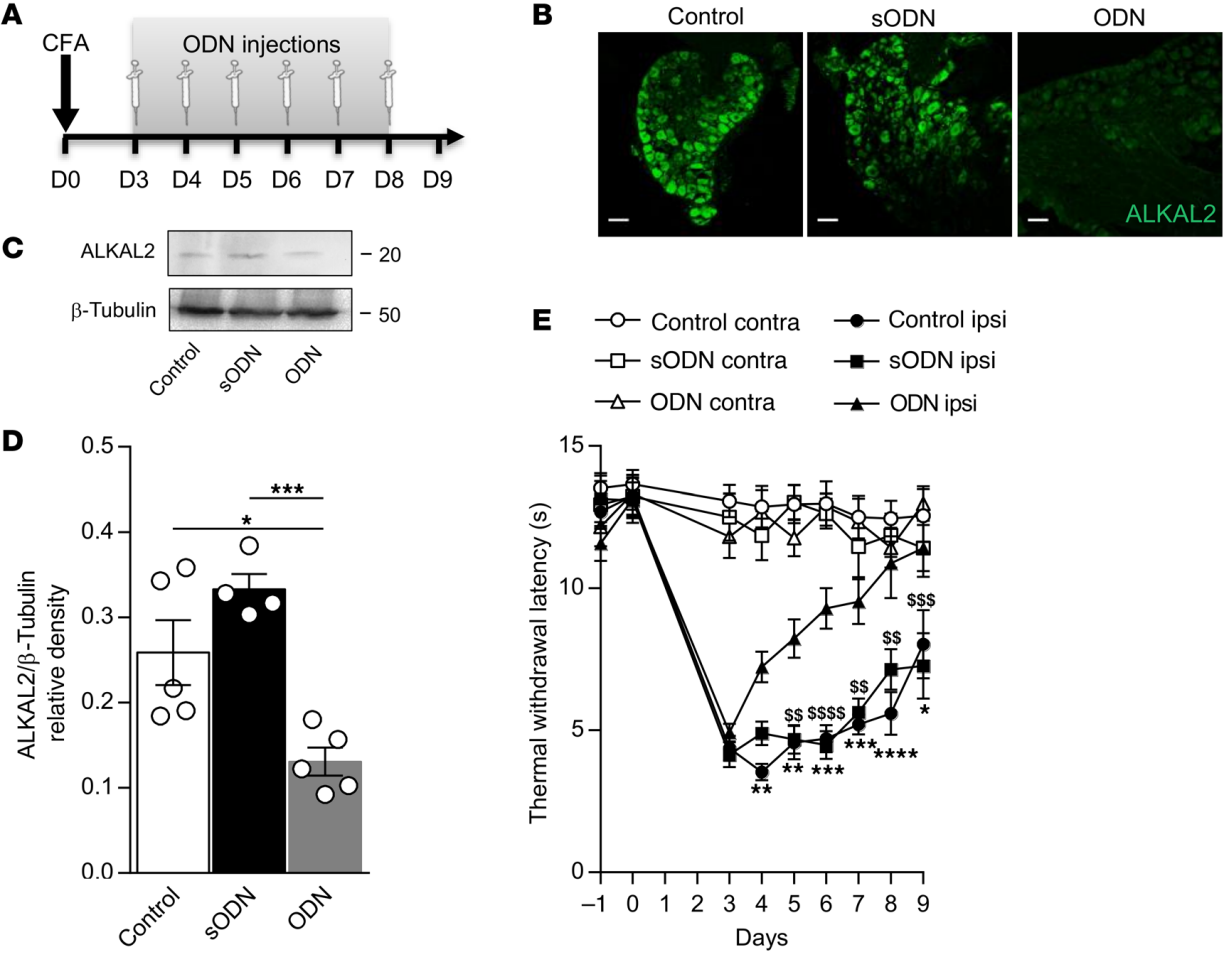
ALKAL2 gene depletion reverses inflammatory pain. (**A**) Schematic illustrating the experimental protocol of ALKAL2 oligodeoxynucleotide injection in the CFA pain model. (**B**) Representative confocal images of ALKAL2 immunostaining in DRG sections from control, ALKAL2, or scrambled ODN–treated animals. Scale bars: 50 μm. (**C**) Western blot of ALKAL2 in lumbar DRG lysates at D9 following injection of ALKAL2 or scrambled ODN, compared with naive control mice. (**D**) Bar graph illustrating the reduction in ALKAL2 protein expression in the ODN-treated animals (*n* = 4–6 mice per group). Statistical analysis was performed using 1-way ANOVA followed by Tukey’s post hoc test. *P < 0.05; ***P < 0.001. (**E**) Measure of thermal withdrawal latency in contralateral and ipsilateral hind paws of CFA-injected animals that received saline control (*n* = 8), scrambled (*n* = 7), or ALKAL2 ODN (*n* = 8). Statistical analysis was performed using 2-way ANOVA followed by Tukey’s post hoc test. *P < 0.05, **P < 0.01, ***P < 0.001 ****P < 0.0001 ODN ipsi vs Control ipsi; $$P< 0.01, $$$P < 0.001, $$$$P < 0.0001 ODN ipsi vs sODN ipsi. Data are represented as mean ± SEM.

To determine whether the production of ALKAL2 by TRPV1^+^ primary afferents may lead to the activation of ALK and subsequent inflammatory hyperalgesia, we first depleted ALKAL2 expression locally in the DRGs of CFA-treated mice using ALKAL2 antisense oligonucleotides (ODNs). The i.t. injection of ALKAL2 ODNs between days 3 and 8 of CFA (Figure 8A) significantly reduced ALKAL2 protein assessed at 8 days after injection in lumbar DRG lysates or tissue sections (Figure 8, B–D). The long-lasting depletion of ALKAL2 induced antinociception, which was indicated by a faster resolution of thermal hyperalgesia in ALKAL2 ODN–treated mice relative to scrambled controls (Figure 8E). Together, these results demonstrate the pronociceptive action of ALKAL2 and hence suggest a central role of ALK in persistent pain.

**Figure 9:**
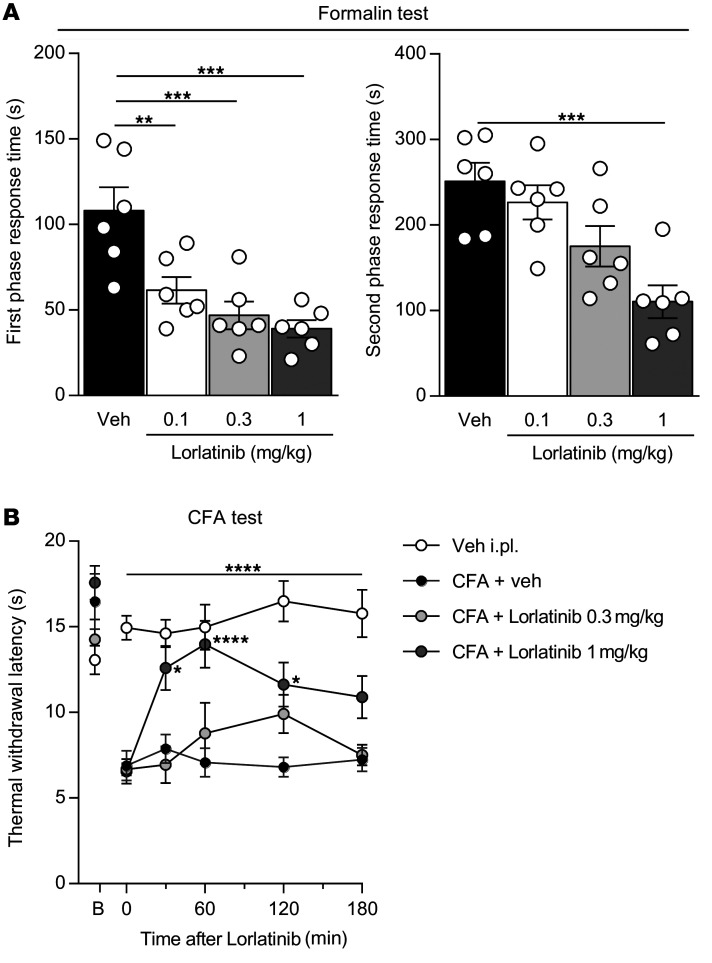
Pharmacological inhibition of ALK receptor induces antinociception in inflammatory pain models. (**A**) Shortening of the nociceptive behavior duration produced by i.pl. formalin (20 μl of 1.25% solution) after administration of increasing doses of lorlatinib by gavage; vehicle was administered as control (*n* = 6 per condition). Statistical analysis was performed using 1-way ANOVA followed by Dunnett’s post hoc test. **P < 0.01; ***P < 0.001 versus vehicle. (**B**) Paw withdrawal latency in response to a thermal stimulus in the CFA model showed the antinociceptive effect of lorlatinib (PBS, *n* = 10; CFA+vehicle, *n* = 10; CFA+lorlatinib, 0.3 mg/kg, *n* = 12; CFA+lorlatinib, 1 mg/kg, *n* = 10). Statistical analysis was performed using 2-way ANOVA followed by Tukey’s post hoc test. *P < 0.05; ****P < 0.0001 versus CFA+vehicle. Data are represented as mean ± SEM.

We next investigated whether blocking ALK receptors in vivo was able to alleviate persistent pain induced by inflammation or nerve injury. A wide range of ALK inhibitors have proven to be safe in clinical trials and are now used in the treatment of malignancy (21–23). We started with the formalin test, which captures mechanisms that are relevant to many clinical pain conditions, including the poorly localized burning and throbbing pain sensation (33). The formalin test triggers 2 phases of nociceptive behaviors; the first is directly linked to the stimulation of the primary sensory neurons, which is followed by a second phase associated with inflammation-induced sensitization. As shown in Figure 9A, the orally available ALK inhibitor lorlatinib significantly inhibited nociceptive scores in a dose-dependent manner in both phases of the formalin test (78% ± 3% in the first phase versus 56% ± 4% in the second phase, *n* = 6). Notably, the maximum dose used was only 1 mg/kg, which is well below the therapeutic dose of 100 mg used for the treatment of malignancies (21).

Next, using the CFA model of chronic inflammatory pain, we assessed the antinociceptive effect of lorlatinib. The highest dose of lorlatinib (1 mg/kg) induced a transient but significant increase in paw withdrawal latency (60% ± 5%, *n* = 8) rapidly after administration (30 minutes) and lasting 2 hours (Figure 9B), indicating an antinociceptive effect of lorlatinib following CFA inflammation.

**Figure 10:**
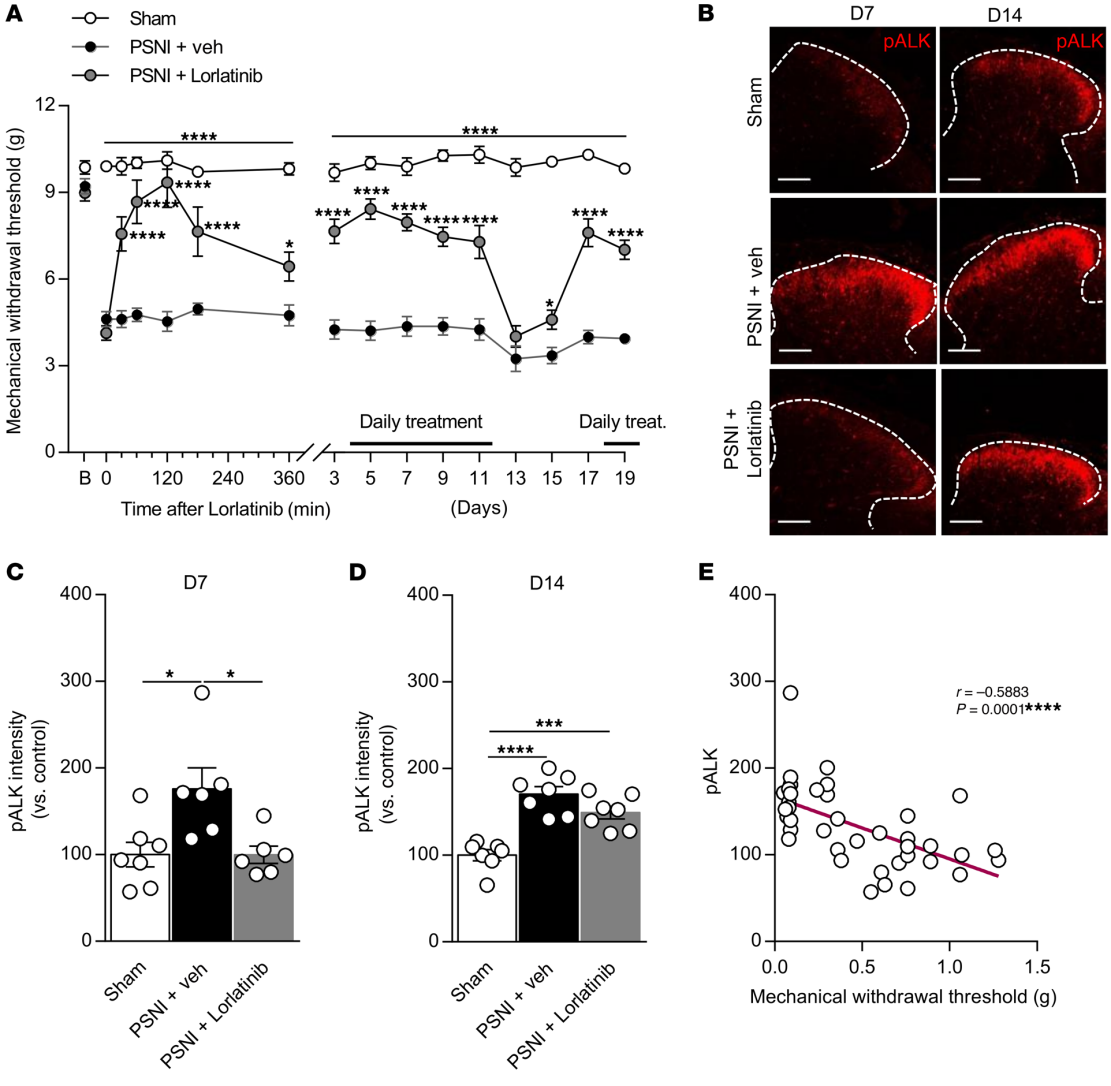
Pharmacological inhibition of ALK receptor induces antinociception in neuropathic pain models. (**A**) PWT to mechanical stimuli was evaluated for 19 days after nerve injury. Lorlatinib inhibited PWT when compared with vehicle only (sham, *n* = 10; PSNI+vehicle, *n* = 10; PSNI+lorlatinib, *n* = 10). Statistical analysis was performed using 2-way ANOVA followed by Tukey’s post hoc test. *P < 0.05; ****P < 0.0001 versus PSNI+vehicle. (**B**) Representative confocal images illustrating the pALK induction in the lamina I and II of the spinal dorsal horn following PSNI. Activation of pALK is normalized by administration of lorlatinib at day 7 and reversed after stopping lorlatinib treatment at day 14. Scale bars: 100 μm. (**C** and **D**) Bar graphs of the pALK signal intensity represented in **B** in the spinal dorsal horn at day 7 (**C**) and day 14 (**D**) (sham, *n* = 7; PSNI+vehicle, *n* = 6–7; PSNI+lorlatinib, *n* = 6–7). Statistical analysis was performed using 1-way ANOVA followed by Tukey’s post hoc test.*P < 0.05; ***P < 0.001; ****P < 0.0001. (**E**) Negative correlation between activation of ALK and PWT. Statistical analysis was performed using Pearson’s test. ****P < 0.0001. Data are represented as mean ± SEM.

Finally, we used the partial sciatic nerve injury (PSNI) model to test the ALK inhibitor on mechanical allodynia. As previously described, PSNI markedly decreased paw withdrawal thresholds (PWTs) to mechanical stimuli relative to those of sham-treated mice, and this tactile allodynia lasted throughout the entire testing period (19 days) (Figure 10A). Importantly, lorlatinib increased the PWT within 30 minutes of daily administration, yet tactile allodynia was restored when the daily treatment was stopped, demonstrating an absence of tolerance to lorlatinib in neuropathic pain conditions (Figure 10A). Next, we assessed the activation of the ALK receptor in the neuropathic pain model. Ligature of the sciatic nerve enhanced the pALK signal in the spinal dorsal horn compared with that of the sham-treated control group (Figure 10, B–D). Activation of ALK was dampened by lorlatinib administration at day 7, but not at day 14, when tactile allodynia reappeared in the ipsilateral hind paw (Figure 10, B–D). Importantly, phosphorylation of ALK was negatively correlated with mechanical withdrawal threshold (Figure 10E), indicating that the level of ALK activation could be used as a surrogate marker of persistent neuropathic pain.

To confirm the effect of ALK inhibition in the spinal dorsal horn, we also employed crizotinib via i.t. delivery in the formalin test and the CFA pain model. Crizotinib showed a similar pattern of antinociceptive properties, blocking nocifensive behaviors evoked by formalin and thermal hyperalgesia after CFA. However, in the formalin test, crizotinib potency was comparable for the early and later phases (76% ± 2% in the first phase versus 81% ± 7% in the second phase, *n* = 8); (Supplemental Figure 6A). Paw withdrawal latency in the CFA model was also inhibited quickly after crizotinib administration, with a robust antinociceptive effect at 30 and 100 ng and a partial reversal of the drug effect at 3 hours after injection (Supplemental Figure 6B).

## Discussion

The lack of treatment options for chronic pain conditions calls for a fundamental reappraisal of the molecular mediators of nociception and sensitization. Recent work indicated the importance of RTKs, including the TrkA and ErbB families (6, 7, 9), in persistent pain conditions. For instance, anti–nerve growth factor (anti-NGF) therapy has proven to be effective in reducing pain in osteoarthritis patients. Furthermore, the clinically approved EGFR inhibitors gefitinib and lapatinib were found to be analgesic in mouse models of inflammatory and neuropathic pain, whereas activation of the EGFR by its ligand epiregulin could enhance pain perception (9, 39). Here, we describe another neuronal RTK that is implicated in hyperalgesic priming. To faithfully capture the inflammation-mediated regulation of gene expression in TRPV1 primary afferents, we took advantage of a pHluorin-tagged TRPV1 knockin mouse rather than a TRPV1-Cre that labels a wider population of TRPV1-lineage neurons (40). We report that ALKAL2, the physiological ligand of ALK, is enriched in TRPV1 nociceptors. Chronic inflammatory pain enhances neuronal ALKAL2 expression and protein levels. Notably, ALKAL2 appears to be restricted to the peripheral nervous system, as low level of proteins were found in different brain regions, including the cortex, hippocampus, and basal ganglia. In cultured DRG neurons, we demonstrated that ALKAL2 reprogrammed TRPV1 nociceptors in an ALK-dependent fashion. This transcriptional regulation leads to morphological and phenotypic changes highlighted by a slight shift in the threshold of spike initiation (not statistically different) and an increase in AP frequency. Importantly, lorlatinib suppressed the hyperexcitability of ALKAL2-sensitized neurons, suggesting a beneficial effect of ALK tyrosine kinase inhibitors in hyperalgesic priming. Finally, the observed effects of ALKAL2 on cultured DRG neurons indicated that inflammation-induced production of ALK ligand may in turn engage ALK signaling.

Although our work uncovered a role of ALKAL2 in neuroplasticity and sensitization of primary afferent neurons, our study does not address what mediators or receptor pathways drive ALKAL2 expression following CFA-induced inflammation. Several pattern recognition receptors transducing pathogen-associated molecular patterns (PAMPs) have been found in DRGs (41, 42). Among them, TLR4 activated by LPS, TRL3, and STING, receptors of bacterial and viral nucleic acids, or TLR5 that binds flagellin may directly or indirectly (via innate immune cells) drive ALKAL2 expression in cutaneous peptidergic nerves. Along these lines, recent studies reported an upregulation of ALKAL2 expression in methyl mercury–induced neurotoxicity (43), which might point to ALKAL2 as a key alarmin molecule produced by nociceptors to regulate not only nociception, but also other physiological functions that could extend to cellular metabolism or stress response.

To determine whether activation of ALK could contribute to pathological pain, we used crizotinib and lorlatinib in nociceptive and neuropathic pain models. We showed that i.t. crizotinib at low doses suppressed nocifensive behaviors in the formalin test. In the CFA model, thermal hyperalgesia caused by hind paw inflammation was significantly reduced. Using lorlatinib, we found a transient but robust antinociceptive effect, even at 1 mg/kg, below the recommended dose for the treatment of ALK-positive metastatic lung cancer, which is usually administered once daily at 100 mg in humans (44). ALK-positive cancers are characterized by gene fusion that induces overexpression or constitutive receptor activation (16). However, ALK activation in the nervous system may preferentially rely on neuronal ALKAL2. This supports the idea that, at least in adults, the dose of ALK inhibitor for pain conditions could be lower than what is used for treating ALK-positive cancer. These findings identify the ALKAL2/ALK axis as a central signaling hub through which inflammation or injury induces nociceptive sensitization.

Previous work identified 2 ALK ligands, ALKAL1 and ALKAL2, that bind to the extracellular domain of the ALK receptor, leading to activation of downstream signaling in cell culture models (26). Interestingly, the 2 ligands display approximately 47% similarity with one another, but not with other mammalian proteins (20–22), making the inhibition of ALKAL2/ALK interaction a potential specific therapeutic approach that might have limited adverse side effects. ALKAL2, also named augmentor-α (AUG-α) or FAM150b, was first described in the adrenal gland (26) and the retina (45), where its signaling through ALK and LTK receptors has been implicated in autoimmunity, neurodevelopment, and cancer. Previous studies also reported that ALKAL2 was able to activate ALK in vivo and coexpression of ALKAL2 with ALK was found to promote neurite outgrowth in neuroblastoma cell lines (35). Here, we show that ALKAL2/ALK signaling induced neurite elongation and branching in DRG neurons, pointing toward receptor targeting as a way to prevent neuroplastic changes associated with peripheral and central sensitization. Functional examination of DRG neurons by electrophysiology revealed that ALKAL2/ALK signaling increases neuronal excitability, thus likely eliciting sensitization at synapses between TRPV1 C-fibers and dorsal horn neurons. Further work will define whether ALK alters neuronal excitability through transcriptional or posttranslational regulation of voltage-gated dependent channels, as previously described with IGF receptor modulation of T-type calcium channels (36).

Prior to the identification of ALKAL2, pleiotrophin and midkine have been proposed to act as ALK ligands (46, 47). Interestingly, these secreted growth factors induce neurite outgrowth and mitogenic activity in fibroblasts and epithelial and endothelial cells, raising questions about the potential role of ALK signaling in tissue inflammation and repair. Accordingly, findings from Zeng et al. have highlighted ALK in regulating the inflammatory signaling pathway of sepsis (39). While future studies are warranted for determining whether neuronal ALKAL2 participates in the inflammatory response, it is plausible that, upon activation, peptidergic TRPV1 nerve endings release ALKAL2 at the periphery, thus contributing to skin and mucosal host responses to infection or injury. ALK was first described as an oncogene, and several of its mutations have been linked to tumorigenesis, including neuroblastomas and non–small cell lung cancer (48), highlighting its importance in cancer biology. Notably, ALK is a member of the insulin receptor superfamily (14) that also promotes neuronal differentiation and maturation. It is expressed throughout the nervous system, particularly during embryogenesis, which supports a role of the receptor in neurodevelopment (13–15, 29). Surprisingly, ALK-deficient mice do not present major behavioral deficits besides thinness due to decreased triglyceride levels and resistance to diet-induced obesity (49). Our present findings point to the importance of ALKAL2/ALK signaling in nociception and provide further knowledge of the biological role of ALK.

Given the relationship between the sympathetic and nociceptive systems, it will be important to determine whether upregulation of ALKAL2 in the DRG neurons promotes sympathetically maintained pain (50) in the context of arthritis or neuropathic pain. Although the ALKAL2 sequence predicts a secreted protein, the modality of action and secretion of this neuronal factor remain to be elucidated. While our data show an activation of ALK in the spinal dorsal horn, suggesting a release of ALKAL2 through axonal transport in the C fibers, one can speculate that ALKAL2 is secreted at the cell soma to act on neighboring neurons and satellite glial cells in the DRG. Finally, ALKAL2 may be released in an activity-dependent manner in the skin and mucosal tissues that receive C-fibers.

Together, our data identified ALKAL2 as a master regulator of injury-induced peripheral sensitization. Our findings are consistent with a model wherein inflammation promotes the expression and secretion of ALKAL2 from TRPV1 nociceptors. ALKAL2 activates ALK to elicit neuronal hyperexcitability along with neurite outgrowth. This phenotypic change drives persistent pain associated with inflammation or nerve injury. Inhibition of ALKAL2/ALK signaling prevents nociceptor hyperexcitability, thereby suppressing pALK in the spinal dorsal horn and alleviating thermal hyperalgesia and mechanical allodynia. Therefore, our work identifies ALK as a future candidate receptor for pain management. As clinical studies have indicated that crizotinib or ceritinib therapy could reduce chest, arm, and shoulder pain in non–small cell lung cancer patients (51–53), it will be important to confirm that ALK inhibition is analgesic in human patients.

## Methods

### Animals.

We used adult male C57BL/6 J mice (6 to 8 weeks old) purchased from the Jackson Laboratory (catalog 664). The TRPV1-pHluorin mice were generated and bred at the University of Calgary Animal Resource Center. Ecliptic GFP (superecliptic pHluorin or EcGFP) was inserted into the trpv1 gene at position 1842, between the 615th and 616th residues (histidine and lysine, respectively) (37), using CRISPR/Cas9-mediated homology–directed repair. Cas9 nuclease was guided to the sequence GCAGATCCCCGACACTTGTG, which was cloned in the pX330 plasmid. The hCas9 sequence from pX330 was cloned in the RCIscript-Goldy TALEN plasmid, replacing the sequence between xenopus globin 5′ and 3′ UTR. Both sgRNA and Cas9 mRNA were prepared by in vitro transcription, using the MEGAshortscript Kit and the mMESSAGE mMACHINE T3 Transcription Kit (Life Technologies), respectively. Single-stranded donor DNA, with a 725 nt upstream and 850 nt downstream homology arm, was prepared using nicking endonucleases. A mixture of sgRNA, Cas9 mRNA, and single-stranded donor DNA was injected into pronuclei of fertilized mouse eggs. PCR and sequence analysis of the resulting mice identified a single mouse as the carrier of correctly inserted EcGFP.

All experiments were conducted on male age-matched animals. Animals were housed at a maximum of 3 per cage (30 × 20 × 15 cm), with water and food ad libitum. They were kept in controlled temperatures of 23 ± 1°C on 12-hour light/12-hour dark cycles (lights on at 7:00 am), and all experiments were performed between 10 am and 3 pm. Different cohorts of mice were used for each test. To reduce the risk of bias that can be caused by an awareness of group assignment, single-blind experiments were performed on animals.

### Human DRG and spinal cord samples.

Human DRG and spinal cord were obtained from 3 brain-dead organ-donor patients (61 to 76 years old). The 3 patients died from stroke. Body temperature was lowered with ice, and blood circulation was maintained for 3 hours before vertebral bloc removal. After organ removal for transplantation purpose, a spinal segment from thoracic level (T9) to the caudal end was removed in 1 piece, and spinal cord and DRGs were immediately dissected in ice-cold, oxygenated HBSS solution. Tissues were subsequently flash frozen in liquid nitrogen. This short time interval allowed good preservation of the tissue, as indicated by the near absence of morphologically altered cells, as previously reported (54).For subsequent in situ hybridization experiments, frozen ganglia in 12 μm sections were prepared with a cryostat and mounted on SuperFrost Plus (Thermo Fisher Scientific).

### RNAScope in situ hybridization.

RNAScope in situ hybridization multiplex chromogenic assay was performed per instructions by Advanced Cell Diagnostics (ACD). Snap-frozen human DRG and spinal cord tissues were cryosectioned at 10 to 14 μm, mounted on SuperFrost Plus slides (Fisher Scientific), and stored at –80°C. The next day, slides were removed from the –80°C freezer and immediately washed with PBS (pH 7.4; 5 minutes, twice), fixed with 4% PFA-PBS, and then dehydrated in 50% ethanol (5 minutes), 70% ethanol (5 minutes), and 100% ethanol (5 minutes) at room temperature (RT). Slides were pretreated with H_2_O_2_ 10 minutes at RT and washed twice in distilled water. Then slides were submerged in 1× boiling RNAScope target retrieval reagent for 5 minutes. After target retrieval agent treatment, slides were transfered in distilled water and then washed in 100% ethanol. The slides were air dried briefly, and then boundaries were drawn around each section using a hydrophobic pen (ImmEdge PAP pen; Vector Laboratories). When hydrophobic boundaries had dried, protease III reagent was added to each section and slides were incubated for 20 minutes at 40°C in a HybEZ oven (ACD). This last step was repeated once, and slides were washed with distilled water before RNAScope assay. The RNAScope assay was performed according to the manufacturer’s instructions using a HybEZ oven (ACD). The probes used were as follows: Hs-Alkal2 (ACD, catalog 588841), Hs-Scn10a (ACD, catalog 406291-C2), and Hs-SCL17A6 (ACD, catalog 415671-C2). At the end of the process, hematoxylin counterstain was performed (30% Gill hematoxylin 1, American Master Tech Scientific/MLS), and slides were mounted with Vectamount mounting medium (Vector Laboratories). Slides were imaged with a Zeiss axio imager (20×).

### Drug treatment, i.t.

The i.t. injections were performed in conscious mice. Briefly, mice were manually restrained, the dorsal fur of each mouse was shaved, the spinal column was arched, and a 30-gauge needle attached in a PE20 polyethylene tube to a 25 μl Hamilton microliter syringe was inserted into the subarachnoid space between the L4 and L5 vertebrae. Accurate positioning of the needle tip was confirmed by a characteristic tail-flick response of animal when the needle was correctly positioned. The i.t. injections of 10 μl were delivered over a period of 5 seconds.

### Formalin test.

Formalin test was performed as originally described (33) and as routinely performed in our lab (55). Briefly, mice were acclimatized in the laboratory for at least 60 minutes before experiments. Animals received 20 μl of formalin solution (1.25 %) prepared in PBS and injected in the plantar surface of the right hind paw (i.pl.). Following i.pl. injections of formalin, mice were immediately placed individually into observation chambers and the time spent licking or biting the injected paw was recorded and considered as a nocifensive response. We observed animals individually and measured nocifensive responses from 0 to 5 minutes (acute nociceptive phase) and 15 to 30 minutes (inflammatory phase). Crizotinib (Sigma-Aldrich) was delivered by i.t. injection 20 minutes prior to testing. Lorlatinib (Sigma-Aldrich) was delivered systemically via intragastric gavage (i.g.) 30 minutes prior to testing.

### Persistent inflammatory pain induced by CFA.

To induce thermal hyperalgesia produced by peripheral inflammation, 20 μl of CFA (Sigma-Aldrich, F5881) was injected s.c. into the plantar surface of the right hind paw (i.pl.) (56). Sham-treated groups received 20 μl of PBS in the ipsilateral paw. Animals were treated with either crizotinib (Sigma-Aldrich) delivered spinally (i.t.), lorlatinib (Sigma-Aldrich) systemically (i.g.), or vehicle (10 ml/kg) 3 days following CFA injection, and their thermal withdrawal thresholds were subsequently tested.

For ALKAL2 depletion, mice were treated with antisense ODN, ALKAL2, scrambled ODN (5 μg, i.t.; Integrated DNA Technologies), or vehicle control for 5 consecutive days at day 3 after CFA injection. Thermal withdrawal threshold was assessed at 3, 4, 5, 6, 7, 8, and 9 days of CFA treatment. Sequences were as follows: design no. 1: ASO-1 DNA sequence: 5′-AAGTGCTTGCTGCACTTCGG; design no. 2: ASO-2 DNA sequence: 5′-GATGGTGCAGTCTCTCGTGT; design no. 3: ASO-3 DNA sequence: 5′-TGGTGTGTCGCTCCTTTGCA; scrambled ODN: 5′-TGTGCTGCTTGTACTGGCCT.

### PSNI-induced neuropathic pain.

Mice were anesthetized with isoflurane (5 % induction, 2.5 % maintenance). A partial ligation of the sciatic nerve was performed by tying the distal one-third to one-half of the dorsal portion of the sciatic nerve, according to the procedure described (57). In sham-operated mice, the sciatic nerve was exposed without ligation. The wound was closed and covered with iodine solution. Fourteen days after surgery, mice were treated with lorlatinib (Sigma-Aldrich) (1 mg/kg, i.g.) or vehicle, while sham-operated animals received only vehicle (10 ml/kg, i.g.). Mechanical withdrawal thresholds were evaluated immediately before the surgeries (baselines), then 14 days after the surgeries (day 0) and at various time points (0.5, 1, 2, 3, 4, 6 hours) after treatment and every 2 days afterwards.

### Thermal hyperalgesia.

Thermal hyperalgesia was determined by measuring the latency to withdrawal of the right hind paws in response to a focused beam of radiant heat (IR = 30) of a Plantar Test apparatus (UgoBasile). Animals were placed individually in a small, enclosed testing arena (20 cm × 18.5 cm × 13 cm, length × width × height) on top of a wire mesh floor. Mice were allowed to acclimate for a period of at least 90 minutes. The device was positioned beneath the animal, so that the radiant heat was directly under the plantar surface of the ipsilateral hind paw. Three trials for each mouse were performed. The apparatus was set at a cut-off time of 30 seconds to avoid tissue damage. Thermal hyperalgesia was evaluated immediately prior to the treatments (time 0) and 15, 45, 90, and 180 minutes after i.t. treatment with crizotinib (Sigma-Aldrich) or after 30, 60, 120, and 180 minutes of lorlatinib (Sigma-Aldrich) administration. For ALKAL-2–induced hyperalgesia, mice were treated with ALKAL2 (MyBioSource LLC, MBS1425538) (0.01; 0.1; 1 μM, i.t.) or vehicle after lorlatinib administration (Sigma-Aldrich) (1 mg/kg, i.g., 30 minutes prior to ALKAL2 administration), and thermal hyperalgesia was evaluated 1, 3, 6, 24, 48, and 72 hours after ALKAL2 treatment.

### Evaluation of mechanical hyperalgesia.

Mechanical hyperalgesia was measured using a dynamic plantar aesthesiometer (DPA; Ugo Basile), as routinely performed in our laboratory (55, 58). Animals were placed individually in small, enclosed testing arenas (20 cm × 8.5 cm × 13 cm, length × width × height) on top of a wire grid platform. Mice were allowed to acclimate for a period of at least 90 minutes. The DPA device was positioned beneath the animal so that the filament was directly under the plantar surface of the ipsilateral hind paw. Each paw was tested 3 times per session.

### Isolation of DRG neurons.

DRG neurons were harvested from adult mice and enzymatically dissociated in HBSS containing 2 mg/ml collagenase type I and 4 mg/ml dispase (both from Invitrogen) for 45 minutes at 37°C. DRGs were rinsed twice in HBSS and once in Neurobasal A culture medium (Thermo Fisher Scientific) supplemented with 2% B-27, 10 % heat-inactivated FBS (HI-FBS), 100 μg/ml streptomycin, 100 U/ml penicillin, 100 ng/ml NGF, and 100 ng/ml glial cell–derived neurotrophic factor (GDNF) (all from Invitrogen). Individual neurons were dispersed by trituration through a fire-polished glass Pasteur pipette in 4 ml media and cultured overnight at 37°C with 5% CO_2_ in 96% humidity on glass coverslips previously treated with 25% poly-ornithine and laminin (both from Sigma-Aldrich).

For coculture experiments, HEK 293T cells (ATCC) were grown to 80% confluence at 37°C (5% CO_2_) in DMEM (+10% FBS, 200 units/ml penicillin, and 0.2 mg/ml streptomycin; Invitrogen) in the Transwell of a 12-well cell culture plate (Gibco, Thermo Fisher Scientific). Cells were transfected with 0.5 or 1 μg of ALKAL2 plasmid (Genomics, ABIN3292379) using the calcium phosphate method and washed 12 hours after transfection. After another 8 hours, isolated DRG neurons plated on glass coverslips were placed in the bottom chamber of the 12-well plate and the media was changed to Neurobasal A culture medium (see above). Electrophysiological recordings or immunohistochemistry were conducted 24 hours later.

### FACS analysis.

At day 3 of CFA, ipsilateral and contralateral DRG tissues (L4–L6) from TRPV1-pHluorin mice were collected and digested separately. After digestion, cells were filtered through a 90 mm mesh (Sarstedt) and washed in PBS 1% FBS. Cells were analyzed on a FACSAria II (BD Bioscience).

### Gene expression analysis.

After FACS sorting, RNA was extracted separately from GFP-positive and GFP-negative cells using an RNeasy Mini Kit (QIAGEN) and eluted in 20 μl of water with the eluate passed twice through the column to increase yield. The quantity of RNA was determined using a Nanodrop 2000c spectrophotometer (Thermo Fisher Scientific). Three biological replicates for the GFP-positive and GFP-negative samples (at ~50 ng/sample) were submitted to the Centre for Applied Genomics (Toronto, Canada). Here, the quality of the samples was assessed using the Agilent Bioanalyzer 2100 with the RNA Pico Chip kit (Agilent Technologies). RNA integrity number values between 6.5 and 7 were achieved. Expression profiling was performed according to the manufacturer’s instructions with Affymetrix GeneChip Mouse Gene 2.0 ST Array. Primary data analysis was carried out with the Affymetrix Expression Console, version 1.4.1.46, software, including the Robust Multiarray Average module for normalization. Gene expression data were log transformed. A change was considered significant when the FDR-corrected *P* value/*q* value thresholds met the criterion *q* < 0.01 at fold changes greater than 2 (expression increments or declines larger than 2). Raw sequence data were deposited in the NCBI’s Gene Expression Omnibus (GEO GSE201227).

### Single-cell RNA-Seq analysis from published data set.

To analyze ALKAL2 (Fam150b) expression in peripheral sensory neurons, the Adolescent, Level 6, Taxonomy Level 2 Pns neurons data set from the mouse brain atlas (35) was extracted and processed using the Seurat package (version 4.1.0) (59) in R (version 4.1.1). Loom files were converted into Seurat object using the LoadLoom function from the SeuratDisk package. Data were then processed with the classical Seurat pipeline. The expression matrix for each population was computed with the AverageExpression function, and the heatmap was generated using the DoHeatmap function of the mean expression matrix of the list of gene of interest.

### Biochemistry.

Collected organs were homogenized using a bullet blender (Next Advance) with SSB02 beads (Next Advance) and lysed in RIPA buffer (0.1% SDS, 1% Triton X-100, and 0.5% Na deoxycholate in PBS; all from Sigma-Aldrich) with Halt protease and phosphatase inhibitors (Thermo Fisher Scientific) for 45 minutes. Lysates were centrifuged at 10,000*g* for 10 minutes at 4°C, supernatants were collected, and protein concentration was quantified and normalized using a Bradford assay (Bio-Rad Laboratories). Total lysates were separated by SDS-PAGE (7%–10%) and transferred onto nitrocellulose membranes (Sigma-Aldrich). Membranes were blocked in 5% nonfat dry milk for 1 hour at RT and then probed with a custom anti-ALKAL2 antibody (1:100 dilution in 5% milk: New England Peptides, project 4633, Seq: BU01949) at 4°C overnight. Membranes were then washed 3 times with TBS-T and incubated with HRP-conjugated anti-rabbit antibodies (1:1000; GE Healthcare, catalog NA934V) for 1 hour at RT. Bands were visualized using the Immobilon Western chemiluminescent HRP Substrate (Bio-Rad), and band density was calculated using ImageJ (NIH). Intensity of rabbit anti–β-tubulin III antibody (1:1000 dilution in 5% milk; Sigma-Aldrich, catalog T2200) band was used for normalization among samples).

### RNA extraction and RT-qPCR.

DRGs were harvested at 72 hours of i.pl. CFA injection and dissociated using a bullet blender (Next Advance) with SSB02 beads (Next Advance) in RLT buffer (QIAGEN). Total RNA was extracted using a RNeasy Mini Kit (QIAGEN) according to the manufacturer’s instructions. The quality and quantity of RNA were determined using a Nanodrop 2000c spectrophotometer (Thermo Fisher Scientific). Relative ALKAL2 gene expression (normalized to GAPDH) was determined by qPCR using BrightGreen PCR Master Mix (ABMgood) and a StepOnePlus Real-time PCR detection system (Applied Biosystems). The designed primers for DNA amplification are listed in Supplemental Table 2.

### Immunostaining and confocal microscopy.

Spinal cords and DRG (L4–L6) were collected from CFA-injected mice and were fixed for either 3 hours (DRG) or 24 hours (spinal cords) in 4% paraformaldehyde (PFA) (Sigma-Aldrich), followed by 24 hours of treatment with 30% sucrose. Tissues were then embedded in OCT (Thermo Fisher Scientific) and cut into 10 μm sections using a cryostat. Tissues were washed 2 times in PBS and then blocked for 60 minutes at RT with a PBS solution containing either 5% BSA or 3% FBS and 0.3% Triton-X 100. Then, tissues were incubated overnight in either PBS 3% BSA or 3% FBS, 0.01% Triton-X 100 at 4°C with either polyclonal chicken anti-GFP (1:500, Invitrogen, catalog A10262), polyclonal rabbit anti-GFP (1:500, Chromotek, catalog PABG1) polyclonal rabbit anti-TRPV1 (1:500, Alomone, catalog ACC-030), polyclonal rabbit anti-CGRP (1:1000, Sigma-Aldrich, catalog PC205L), anti–IB4-coupled Alexa Fluor 594 (1:1000, Invitrogen, catalog I21412), polyclonal sheep anti-TH (1:500, Millipore, catalog AB1542), polyclonal goat anti-GFRα3 (1:500, R&D Systems, catalog VFU021721), monoclonal mouse anti-NF200 (1:500, Sigma-Aldrich, catalog N5389), or rabbit anti-ALKAL2 (1:1000, New England Peptide, project 4633, Seq: BU01949). After washing in PBS, tissues were incubated for 1 hour at RT with secondary antibodies (anti-chicken IgG conjugated to Alexa Fluor 488 (Sigma-Aldrich, catalog SAB4600031), anti-rabbit IgG conjugated to Alexa Fluor 488 (Invitrogen, catalogA11008), anti-rabbit IgG conjugated to Alexa Fluor 555 (Invitrogen, catalog A21428), anti-sheep IgG conjugated to Alexa Fluor 555 (Invitrogen, catalog A21099), anti-goat IgG conjugated to Alexa Fluor 488 (Invitrogen, catalog A21467), or anti-mouse IgG conjugated to Alexa Fluor 488(Invitrogen, catalog A11001) for 1 hour at RT. Slides were washed in PBS twice, mounted with Aqua PolyMount (Polysciences Inc.), and imaged on a Zeiss 510 confocal microscope. Image analysis was conducted using ImageJ software as described previously (60).

For the sciatic nerve, nerves were collected and fixed for 24 hours in 4% PFA (Sigma-Aldrich) followed by 24 hours of treatment with 30% sucrose. Tissues were then embedded in OCT and cut in 10 μm sections using a cryostat. Tissues were washed 2 times in PBS and then blocked for 60 minutes at RT with a PBS solution containing 3% FBS and 0.3% Triton-X 100. Then tissues were incubated overnight in PBS 3% FBS, 0.01% Triton-X 100 at 4°C with either rabbit anti-ALKAL2 (1:1000, New England Peptide, project 4633, seq: BU01949), mouse anti-NF200 (1:500, Sigma-Aldrich, catalog N5389), goat anti-GFRα3 (1:500, R&D Systems, catalog VFU021721), or anti-IB4–coupled Alexa Fluor 594 (1:1000, Invitrogen). After washing in PBS, tissues were incubated for 1 hour at RT with secondary antibodies (anti-mouse IgG conjugated to Alexa Fluor 488, Invitrogen, catalog A11001; anti-rabbit IgG conjugated to Alexa Fluor 488, Invitrogen, catalog A11008; anti-rabbit IgG conjugated to Alexa Fluor 555, Invitrogen, catalog A21428; and anti-goat IgG conjugated to Alexa Fluor 488, Invitrogen, catalog A21467). Slides were washed in PBS twice and mounted with Aqua PolyMount (Polysciences Inc.). Slides were imaged on a Zeiss 510 confocal microscope. Image analysis was conducted using ImageJ software.

To evaluate the phosphorylation of ALK in the dorsal horn, mice were perfused with PBS and then PFA 4%. Isolated lumbar (L4–L6) spinal cord sections were dehydrated in 30% sucrose overnight. Tissues were then embedded in OCT and cut into 10 μm sections using a cryostat. Tissues were washed 2 times and then blocked for 60 minutes at RT in TBS solution containing 0.2% Triton-X 100, 0.05% Tween 20 (TBS-T), and 5% goat serum plus 5% donkey serum (Sigma-Aldrich). For pALK immunostaining, we used polyclonal rabbit anti-pALK (1:100, Cell Signaling Technology, catalog 3341) in blocking buffer. After extensive washing in the blocking buffer, tissues were incubated for 1 hour at RT with secondary antibodies (anti-rabbit IgG conjugated to Alexa Fluor 555, Invitrogen, catalog A21428). Slides were washed and mounted with Aqua PolyMount (Polysciences Inc.) and imaged on a Zeiss 510 confocal microscope. Image analysis was conducted using ImageJ software.

### Neurite outgrowth assay.

DRG neurons were cocultured with ALKAL2-expressing HEK cells (see above) in Neurobasal A medium. After 24 hours, the cells were washed twice with HBSS, fixed in 4% PFA for 15 minutes, incubated in blocking solution (PBS+1% BSA) for 30 minutes, and then immunostained with anti–β-tubulin III antibody (rabbit, 1:1000, Sigma-Aldrich, catalog T2200) overnight at 4°C. Cells were washed in PBS twice, then incubated with a goat anti-rabbit IgG conjugated to Alexa Fluor 488 (1:2000, Invitrogen, catalog A11008) for 1 hour. After several washes, coverslips were mounted on slides and confocal images acquired. Alexa Fluor 488 antibody was visualized by excitation with an argon laser (514 nm) and emission detected using a long-pass 530 nm filter.

### Sprouting related to inflammatory pain.

Paw inflammation was induced by s.c. injection of 20 μl of CFA in the plantar surface of the right hind paw. The control group received 20 μl of PBS in the ipsilateral paw. Animals were treated daily with lorlatinib (Sigma-Aldrich) systemically (i.g. 1 mg/kg) or vehicle for 3 days following CFA injection. Paws were collected and were fixed for 24 hours in 4% PFA (Sigma-Aldrich) followed by 24 hours treatment with 30% sucrose. Footpads were then embedded in OCT and cut at 30 μm sections onto SuperFrost slides (VWR International). Tissues were washed 3 times in PBS and then blocked for 60 minutes at RT with a PBS solution containing 5% BSA and 1% Triton-X 100. Sections were incubated overnight in PBS 3% BSA, 0.3% Triton-X 100 at 4°C with rabbit anti-GFP (1:500, Chromotek, catalog PABG1). After washing in PBS, tissues were incubated with secondary antibodies (anti-rabbit IgG conjugated to Alexa Fluor 555; Invitrogen, catalog A21428) for 1 hour at RT. Slides were washed in PBS twice and mounted with Aqua PolyMount (Polysciences Inc.). Images were acquired on a Zeiss 510 confocal microscope. Image analysis was conducted using ImageJ software.

### Electrophysiological measurements.

Electrophysiological recordings were conducted using an external solution containing 140.0 mM NaCl, 1.5 mM CaCl_2_, 2.0 mM MgCl_2_, 5.0 mM KCl, 10.0 mM HEPES, and 10.0 mM d-glucose, pH 7.4, adjusted with NaOH, on the stage of an inverted epi-fluorescence microscope (Olympus IX51). DRG neurons were recorded based on size, considering that TRPV1 is expressed in small neurons (<20μM), and pHluorin fluorescent signal. APs were recorded using current clamp. Borosilicate glass (Harvard Apparatus Ltd.) pipettes were pulled and polished to 2 to 5 MΩ resistance with a DMZ-Universal Puller (Zeitz-Instruments GmbH.) and filled with an internal solution containing 140.0 mM KCl, 5.00 mM NaCl, 1 mM CaCl_2_, 1.0 mM EGTA, 10.0 mM HEPES, 1.0 mM MgCl_2_, and 3.0 mM ATP Na2, pH 7.3, adjusted with KOH. All solutions were prepared and used at RT (22 ± 2°C) and their osmolarity adjusted to 310 mOsm. For the current clamp experiments, the spontaneous activity of the DRG neurons was recorded at RT (~22°C) for 3 minutes before application of ALKAL2 (MyBioSource LLC, MBS1425538) (1 μM applied to the bath at approximately 1000 μm from the cell at a rate of 500 μl/minutes). Only the neurons in which the resting membrane potential was more negative than –40 mV and responded to capsaicin (1 μM) (Sigma-Aldrich) were used. Recordings were performed using an Axopatch 200B amplifier (Axon Instruments). Current-clamp protocols were applied using pClamp, version 10.4, software (Axon Instruments). Data were filtered at 5 kHz (current clamp) and digitized at 10 kHz with a Digidata 1550 A converter (Axon Instruments). Average DRG neuron capacitance was 12.45 ± 0.85 pF. Only the cells that exhibited a stable voltage control throughout the recording were used for analysis.

### Statistics.

For electrophysiology, data analysis and offline leak subtraction were completed in Clampfit, version 10.4 (Axon Instruments). Statistical analysis and graphs were completed using GraphPad Prism 7 software. Data are plotted as mean ± SEM, and numbers in parentheses reflect the number of cells/animals (*n*). Normality distribution was verified using the D’Agostino-Pearson test. For Gaussian data, a paired Student’s *t* test was used to compare data before and after drug treatment and unpaired 2-tailed Student’s *t* test was used to assess statistical significance when comparing 2 means. One-way ANOVA followed by Tukey’s post hoc test was used to compare 3 groups, and 2-way ANOVA followed by Tukey’s post hoc test was used for multiple comparisons. For non-Gaussian data, the nonparametric Mann Whitney *U* unpaired test was used to assess statistical significance when comparing 2 means, and Kruskal-Wallis followed by Tukey’s post hoc test was used to compare 3 groups. A *P* value of less than 0.05 was considered significant.

### Study approval.

Human DRG and spinal cord were obtained from 3 brain-dead organ-donor patients (61 to 76 years old) under the approval of the French institution for organ transplantation (Agence de la Biomédecine, DC-2014-2420).All animal procedures were reviewed and approved by the University of Calgary Animal Care Committee and were in accordance with the international guidelines for the ethical use of animals in research and guidelines of the Canadian Council on Animal Care.

## Author contributions

CA conceived the project. MCI, MD, VMG, L Basso, FA, NSA, AH, RF, JM, VS, GP, NL, FVL, L Bauchet, and PFM designed experiments and methodology. MCI, VMG, L Basso, and MD performed experiments. MD, MC, NSA, L Basso, JM, and EB conducted the revisions. CA, GWZ, and EB acquired funding. CA performed project administration. CA supervised the project. MCI, MD, and CA wrote the original draft of the manuscript. CA, L Basso, GWZ, and EB reviewed and edited the manuscript. MD and CA revised the manuscript.

## Supplementary Material

Supplemental data

## Version 1 05/24/2022

In-Press Preview

## Version 2 06/15/2022

Electronic publication
