# Supplementary material for: The neuronal tyrosine kinase receptor ligand ALKAL2 mediates persistent pain
Source: J Clin Invest. 2022 Jun 15;132(12):e154317. doi: 10.1172/JCI154317 (PMC9197515; doi:10.1172/JCI154317)
Supplement: Supplemental data [file jci-132-154317-s134.pdf]

The neuronal tyrosine kinase receptor ligand ALKAL2 mediates persistent pain.

Manon Defaye, Mircea C. Iftinca, Vinicius M. Gadotti, Lilian Basso, Nasser S. Abdullah, Mélissa Cuménal, Francina Agosti, Ahmed Hassan, Robyn Flynn, Jérémy Martin, Vanessa Soubeyre, Gaetan Poulen, Nicolas Lonjon, Florence Vachiery-Lahaye, Luc Bauchet, Pierre Francois Mery, Emmanuel Bourinet, Gerald W. Zamponi and Christophe Altier
*Corresponding author: altier@ucalgary.ca

This file includes:

Supplemental Figures. 1 to 5
Supplemental Tables 1 to 2

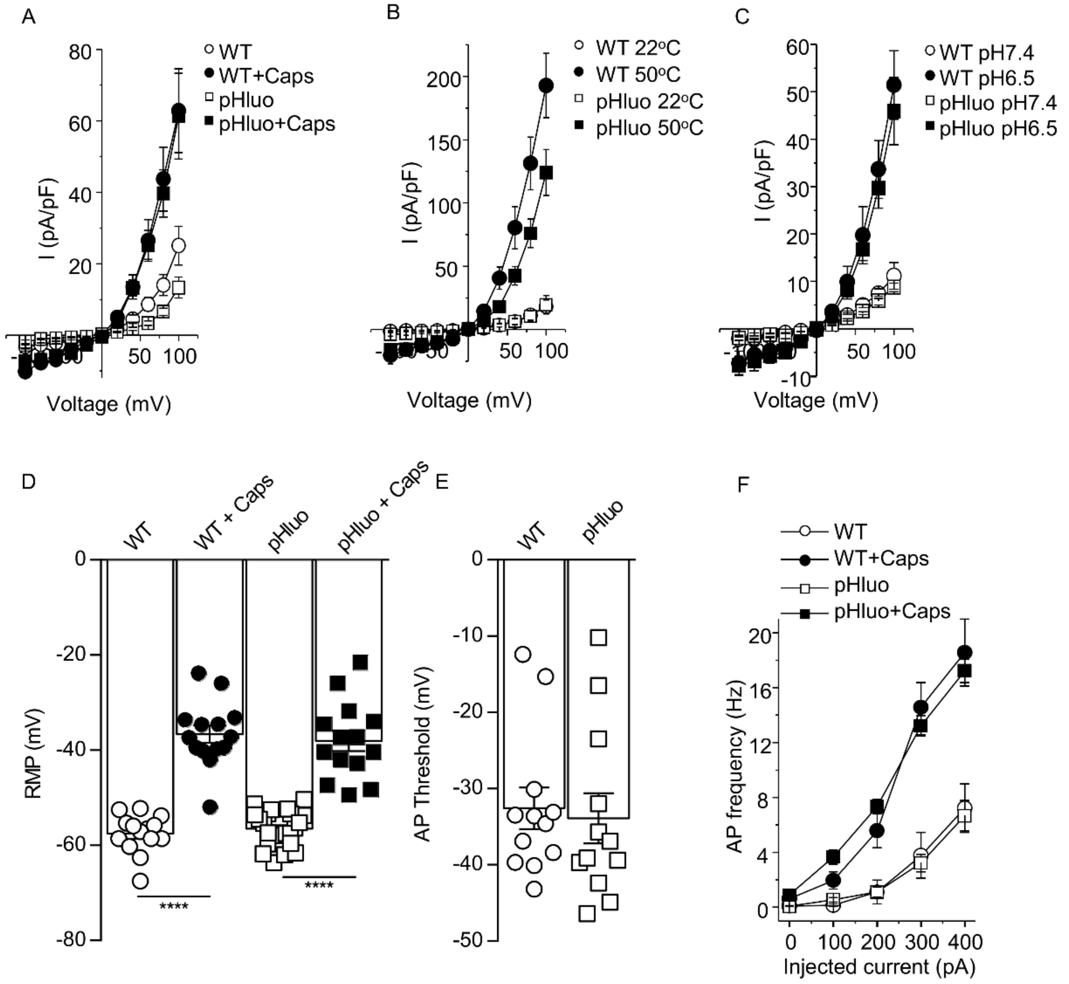

Supplemental Figure 1. Characterization of the TRPV1-pHluorin channel.
(**A**) I-V curves of capsaicin (100nM)-evoked TRPV1 current in HEK cells transfected with either TRPV1 WT (circles, n = 8) or TRPV1-pHluorin (squares, n = 9) obtained from a ramp between -100 mV and +100 mV from a holding potential of 0 mV. (**B**) I-V curves of TRPV1 current recorded from HEK cells transfected with either TRPV1 WT (circles, n = 7) or TRPV1-pHluorin (squares, n = 7) and exposed to either 22^{0}C (control) or 50^{0}C. (**C**) I-V curves of TRPV1 current recorded from HEK cells transfected with either TRPV1 WT (circles, n = 7) or TRPV1-pHluorin (squares, n = 7) exposed to either pH 7.4 (control) or pH 6.5. (**D**) Bar graph showing the resting membrane potential (RMP) in DRG neurons from WT and TRPV1-pHluorin mice at steady state and after exposure to capsaicin (100 nM) (n = 14 per condition). Statistical analysis was performed One-way ANOVA followed by Bonferroni post-hoc (**** $p < 0.01$). (**E**) Bar graph showing the action potential (AP) threshold in DRG neurons from WT and TRPV1-pHluorin mice. (n = 12 per group). Statistical analysis was performed using unpaired t-test. (**F**) AP frequency evoked by 100, 200, 300 and 400 pA current injections (1s) in DRG neurons from WT (circles, n = 9) and TRPV1-pHluorin (squares, n = 11) animals in the absence and presence of capsaicin (100 nM). Results indicate mean ± SEM.

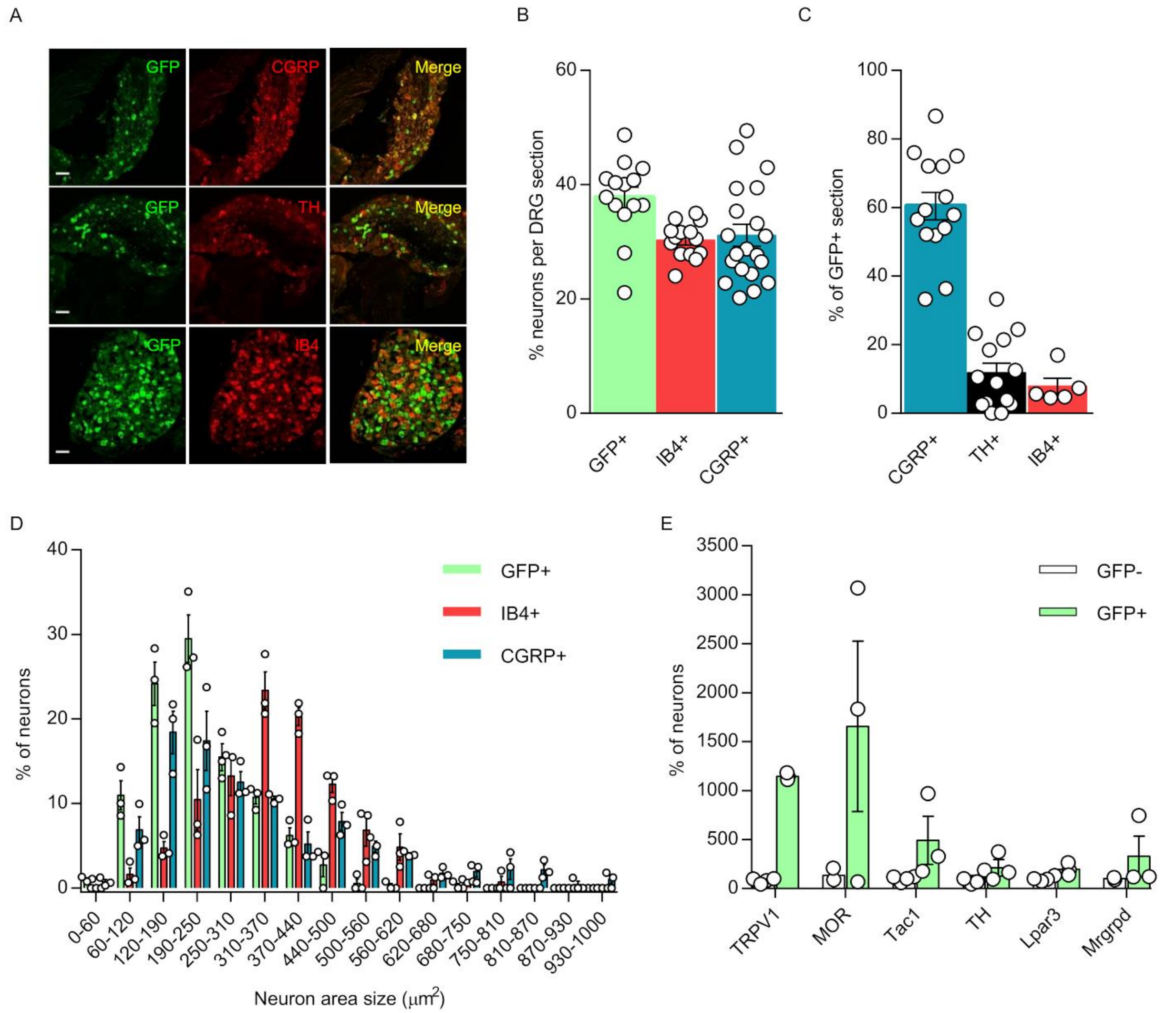

Supplemental Figure 2. Characterization of the TRPV1-pHluorin expressing neurons. (**A**) Co-immunostaining of GFP (left) with markers of peptidergic (Calcitonin gene-related peptide, CGRP), non-peptidergic (Isolectine B4, IB4), C-LTMR (Tyrosine hydroxylase, TH) neurons (middle) in DRG section. Scale bars, 50 µm. (**B**) Bar graph summarizing the results of the percentage of GFP+, IB4+ and CGRP+ neurons in DRG from TRPV1-phulorin mice. (**C**) Bar graph summarizing the results in (**A**). Note that most (~60%) of the GFP+ neurons are peptidergic. (**D**) Neuronal size distribution of DRG neurons positive for GFP, IB4 and CGRP (n = 3). (**E**) qRT-PCR analysis of selected genes (Transient Receptor Potential Vanilloid type 1, TRPV1, Mu-opioid receptors, MOR; Substance P, Tac1; Tyrosine hydroxylase, TH; Lysophosphatidic Acid Receptor 3, Lpar3; MAS Related GPR Family Member D, MrgprD) from GFP+ and GFP- neurons (n = 3-4 GFP-, n = 3 GFP +). Results indicate mean ± SEM.

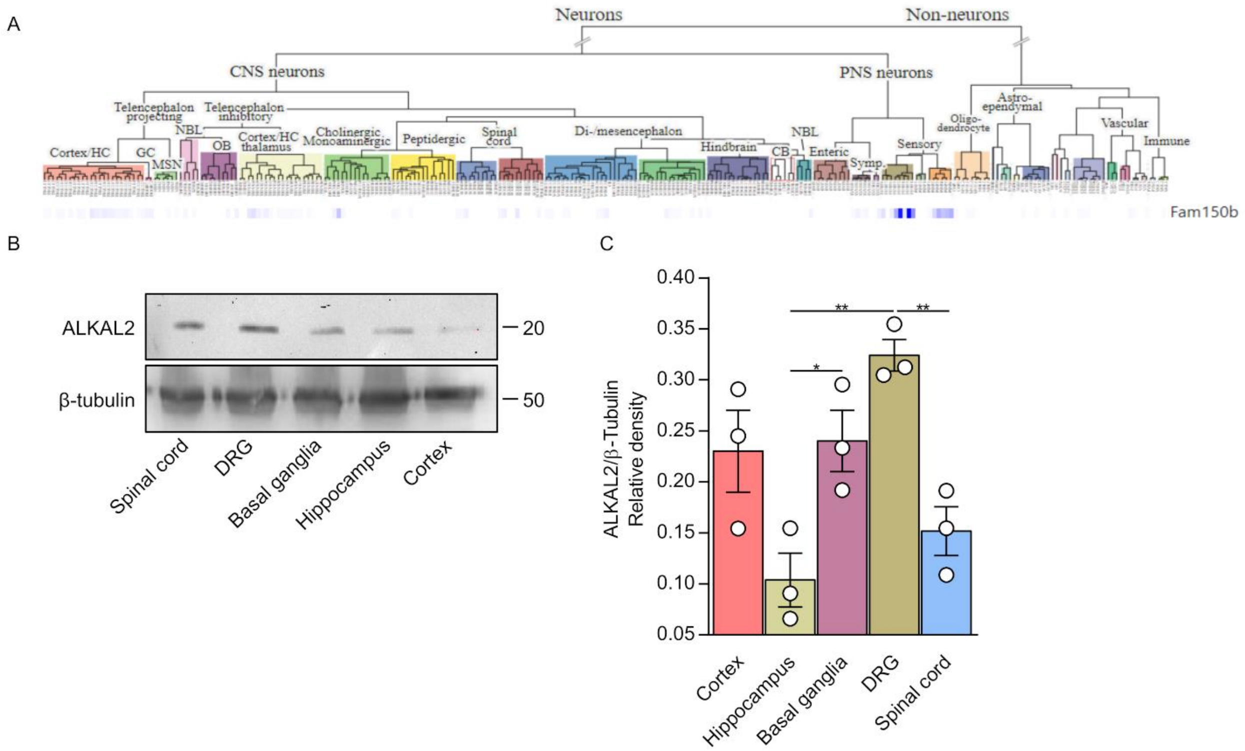

Supplemental Figure 3. ALKAL2 expression in the mouse nervous system.
(**A**) ALKAL2 expression in neuronal and non-neuronal cells of the CNS and PNS, from a mouse single-cell RNA-sequencing dataset (Linnarsson's lab; http://mousebrain.org/genesearch.html). (**B**) Representative western blot showing the level of ALKAL2 protein in the CNS and PNS. (**C**) Quantification of ALKAL2 protein level from the western blot experiments. Each dot represents a sample collected from a different animal (n = 3 per condition). Statistical analysis was performed using One-way ANOVA followed by Tukey post-hoc test (* $p < 0.05$, ** $p < 0.01$). Results indicate mean ± SEM.

A

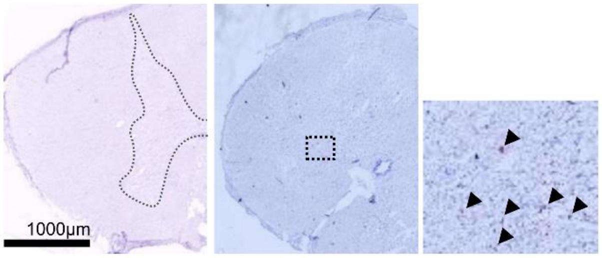

Supplemental Figure 4. ALKAL2 is not found in the human spinal cord.
Representative RNAscope image showing the absence of ALKAL2 expression in human spinal cord. The probes used were Hs-Alkal2 (ACD, # 588841) in light blue, Vglut2 Hs-SCL17A6 (ACD, #415671-C2) in red, as a positive control, and hematoxylin (purple).

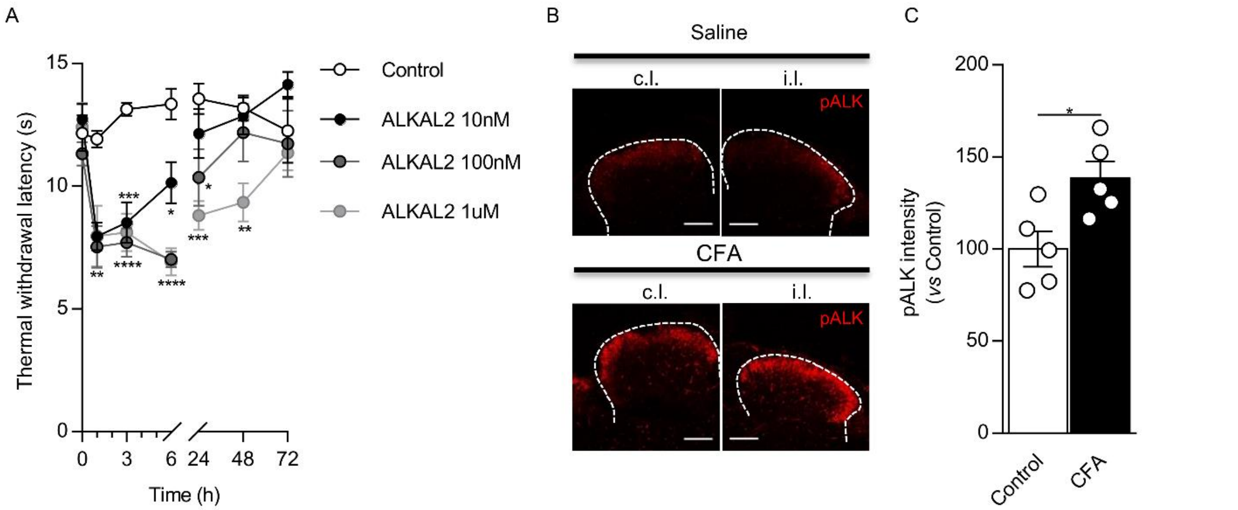

Supplemental Figure 5. ALKAL2 induces pain by activating ALK in the spinal dorsal horn. (**A**) Intrathecal ALKAL2 administration induces thermal hypersensitivity which is back to baseline 72 hours after administration. The time-course of thermal hypersensitivity is dose-dependent (n = 5 for each group). Statistical analysis was performed using Two-way ANOVA followed by Tukey post hoc test (* $p < 0.05$; ** $p < 0.01$, *** $p < 0.001$, **** $p < 0.0001$). (**B**) Intraplantar CFA enhanced pALK signal in the ipsilateral (CFA injected) spinal dorsal horn. (**C**) Bar graph showing the fold change in pALK between ipsilateral and contralateral dorsal horn after intraplantar saline or CFA (n = 5, each condition). Scale bars, 100 µm. Statistical analysis was performed using unpaired t-test (* $p < 0.05$). Results indicate mean ± SEM.

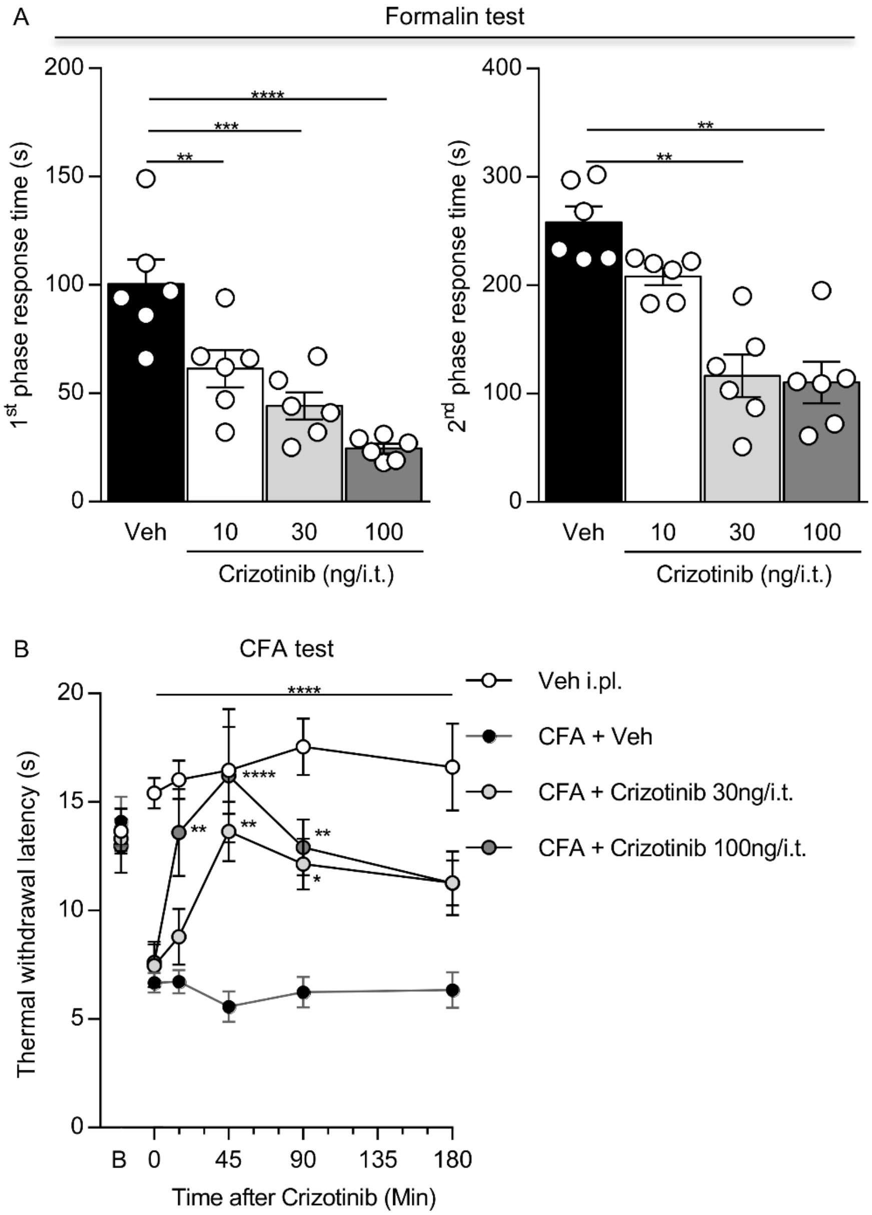

Supplemental Figure 6. Crizotinib reverses acute and chronic inflammatory hyperalgesia. (**A**) Administration of increasing doses of Crizotinib (i.t.) significantly shortened the nociceptive behavior duration after administration of formalin (20 µl of 1.25% solution intraplantar), when compared to vehicle (n = 6 per group). Statistical analysis was performed using One-way ANOVA followed by Dunnett post hoc test (1st phase) or Kruskal-Wallis followed by Dunn's post hoc test (2nd phase) (** p <0.01, *** p <0.001, **** p <0.001 vs Veh). (**B**) The paw withdrawal latency to thermal stimulus, at 3 days post CFA, showed the anti-nociceptive effect of Crizotinib (n = 7 PBS; n = 7 CFA + vehicle; n = 8 CFA + Crizotinib 30 ng and n = 9 CFA + Crizotinib 100 ng). Statistical analysis was performed using One-way ANOVA followed by Tukey post hoc test (A) (** p <0.01, *** p <0.001) and Two-way ANOVA (B) followed by Tukey post hoc test (* p < 0.05, ** p < 0.1, **** p < 0.0001 vs CFA + Veh). Results indicate mean ± SEM.

Supplemental Table 1. Differentially expressed genes. Upregulated and downregulated genes with statistical significance and fold change between ipsilateral (CFA) and contralateral groups. Significant genes were selected by fold change (>2.0 or < –2.0-fold) and adjusted p-value (<0.05).

Upregulated		Downregulated	
**Symbol**	**Fold change**	**Symbol**	**Fold change**
**ALKAL2**	**2**	n-R5s71	-2.06
Wfdc6a	2.06	n-R5s152	-2.2
Vmn1r20	2.04	mt-Ti	-2.18
Trbv13-1	2.02	mt-Ta	-2.13
Trav9n-4	2.13	Vmn1r18	-2.02
Traj53	2.13	Uts2b	-2.07
Tmem173	2.22	Tnnt2	-2.5
Tdpoz2	2.26	Spata31d1b	-2.38
Ssxb9	2.53	Snord82	-2.18
Psg20	2.41	Rps15	-2.39
Olfr784	2.07	Psg22	-2.19
Nlrp5	2.06	Prg4	-2.19
Mptx2	2.05	Pln	-2.18
Mir693	2.23	Orm2	-2
Mir296	2.54	Olfr988	-2.54
Mir1900	2.5	Olfr610	-2.54
Mir1224	2.21	Olfr591	-2.08
LOC101055953	2.01	Olfr59	-2.65
Itk	2.08	Olfr494	-2.15
Igkv4-80	2.42	Olfr467	-2.07
Ighv1-80	4.2	Olfr299	-2.72
Ifnl3	2.02	Olfr1356	-2
Gpx2-ps1	2.42	Nppa	-2.52
Gm9257	2.13	Naip6	-2.61
Gm8212	2.44	Myoz2	-2.05
Gm5862	2.12	Mir501	-2.13
Gm5071	2.14	LOC101056278	-2.11
Ifnl3	2.02	Il1r2	-2.17
Gm25344	2.26	Igkv14-126	-2.48
Gm24984	2.17	Ighv1-20	-2.43
Gm24938	2.08	Igh-VJ558	-2.86
Gm24885	2.21	Gm8247	-2.29
Gm24402	2.81	Gm5565	-2.45
Gm24203	2.21	Gm4963	-2.91
Gm23244	2.9	Gm4825	-2.35
Gm22927	2.29	Gm26251	-2.21
Gm22876	2.47	Gm26036	-2.02
Gm22535	2.13	Gm26016	-2.05
Gm22509	2.51	Gm25408	-2.8
Gm20736	2.27	Gm25102	-2.04
Gm17268	2.02	Gm25008	-3.89
Gm16391	2.18	Gm24282	-2.34
Gm16248	-2.19	Gm24149	-2.3
Gm15182	2.07	Gm24117	-2.29
Gm15127	2.7	Gm24009	-2.48
Gm11677	2.59	Gm23741	-2
Gm11084	2.61	Gm23201	-2.62
Gm10923	2.2	Gm23102	-2.1
Gm10700	2.52	Gm22883	-2.17
Fbxw21	2.15	Gm22831	-2.17
Bdnf	2.26	Gm22749	-2.17
1700093J21Rik	2	Gm22740	-2.02
		Gm22739	-3.07
		Gm22602	-2.35
		Gm22558	-2.21
		Gm22341	-2.1
		Gm22301	-2.02
		Gm22289	-2.18
		Gm22007	-2.4
		Fabp4	-2.02
		Dsg1a	-2.03
		Ctsj	-2.05
		Cma2	-2.02
		C1ql1	-2.04
		Aspn	-2.25
		Apol11b	-3.05
		Adam34	-2.1
		Actc1	-2.52
		4930526F13Rik	-2.09
		n-R5s71	-2.06
		n-R5s152	-2.2

Supplemental Table 2. Primer sequences.

Gene	Primer forward	Primer reverse
ALKAL2	TGAGGTTGCTAGTTGAGCTGG	AGGAACAATCTCCACTCTCTGC
GAPDH	CCATGGAGAAGGCTGGGG	CAAAGTTGTCATGGATGACC
TRPV1	CAACAAGAAGGGGCTTACACC	TCTGGAGAATGTAGGCCAAGAC
TH	CCCAAGGGTTCAGAAGAG	GGGCATCCTCGATGAGACT
MOR	GAGCCACAGCCTGTGCCCT	CGTGCTAGTGGCTAAGGCATC
TAC1	AAAGCAGGAGCTGGGGACTA	TGTTCGATTTTGCGGTCAGA
LPAR3	GTCTTAGGCGCCTTCGTGG	TTGCACGTTACACTGCTTGC
MRGPRD	GGTGCTGCTGGAAACACTTC	TGGAGTTCATTCCTGCTCCTG
